# Supplementary figures and images for: Mathematical modeling of N-803 treatment in SIV-infected non-human primates
Source: PLoS Comput Biol. 2021 Jul 28;17(7):e1009204. doi: 10.1371/journal.pcbi.1009204 (PMC8351941; doi:10.1371/journal.pcbi.1009204)

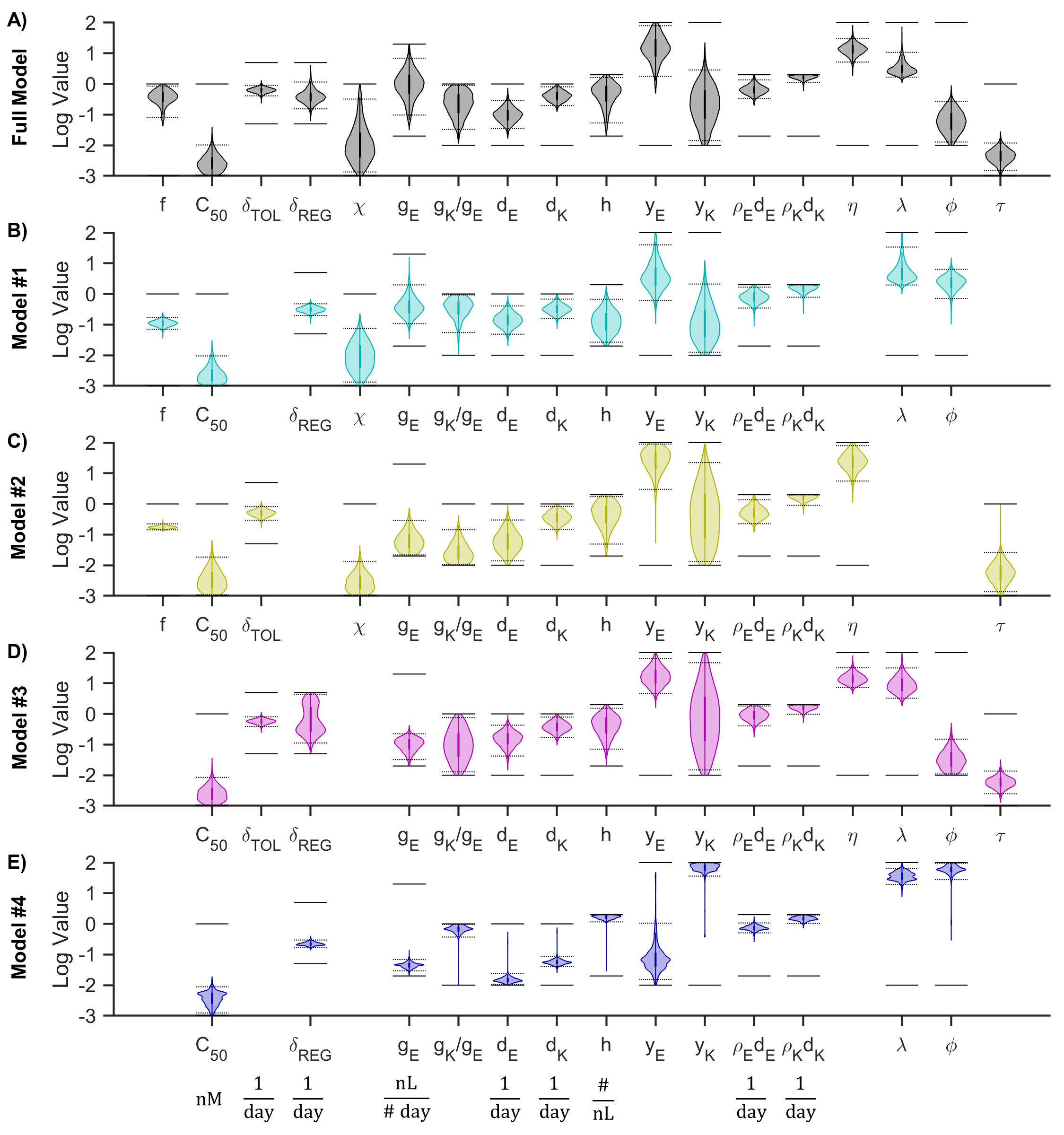

Supplement: S1 Fig — Panels (A-E) show the Bayesian MCMC sample of the posterior distributions of parameter values for the full model and for models #1-4 on a logarithmic scale. Bayesian 95% credible intervals are shown as dotted lines. Allowed parameter ranges (from Table 3) are shown as solid lines. Note that some units of measurement (shown below panel E) are different from those in Table 3. (TIF) [file pcbi.1009204.s001.tif]

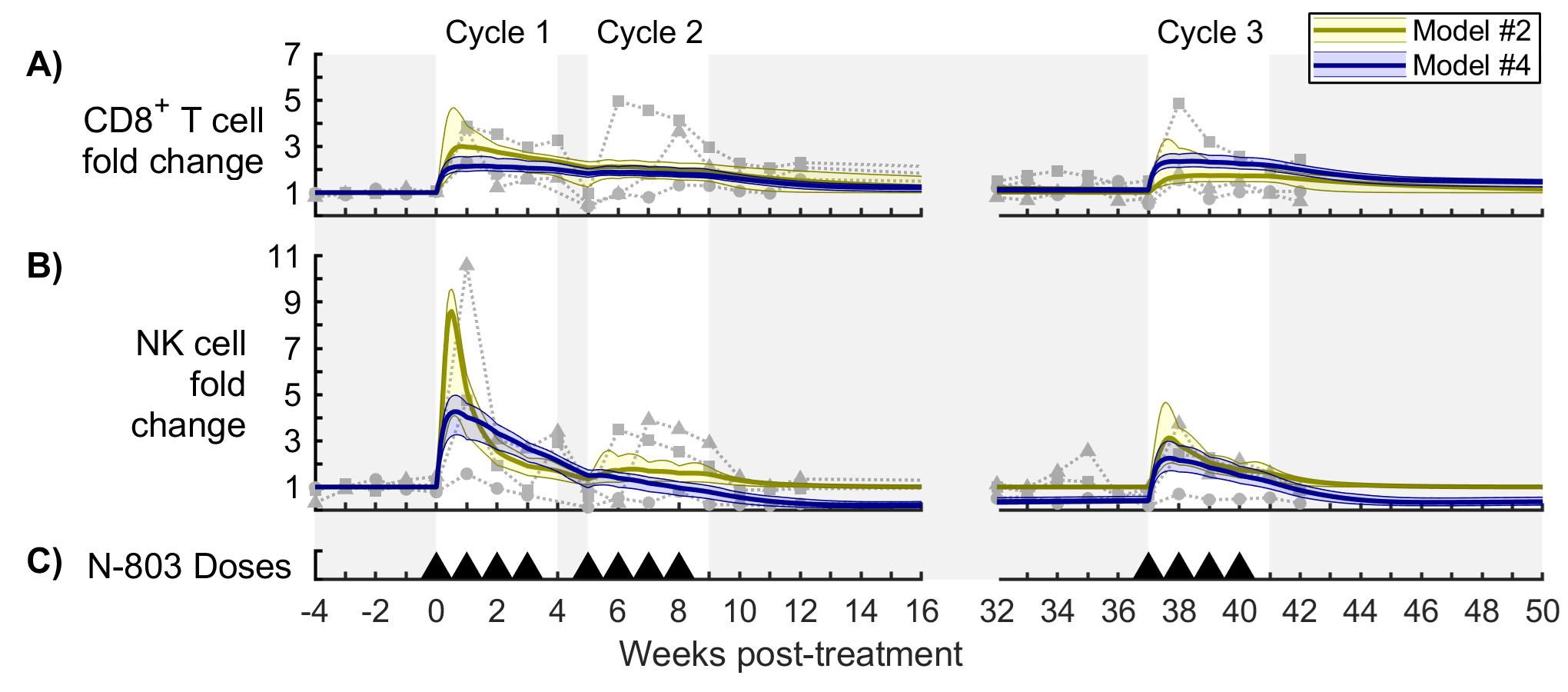

Supplement: S2 Fig — Panels (A,B) show fold change in CD8+ T cells and NK cells in the peripheral blood, respectively, for the model without immune regulation (yellow model #2) and the model without drug tolerance or viral escape (blue model #4). The bold line corresponds to the best-fit model, and the shaded region corresponds to the Bayesian 95% credible interval. See S1 Fig for corresponding parameter distributions. Data from N-803-treated SIV-infected NHPs are shown as different symbols for each NHP [16]. Panel (C) shows timing of 0.1 mg/kg subcutaneous doses of N-803. (TIF) [file pcbi.1009204.s002.tif]

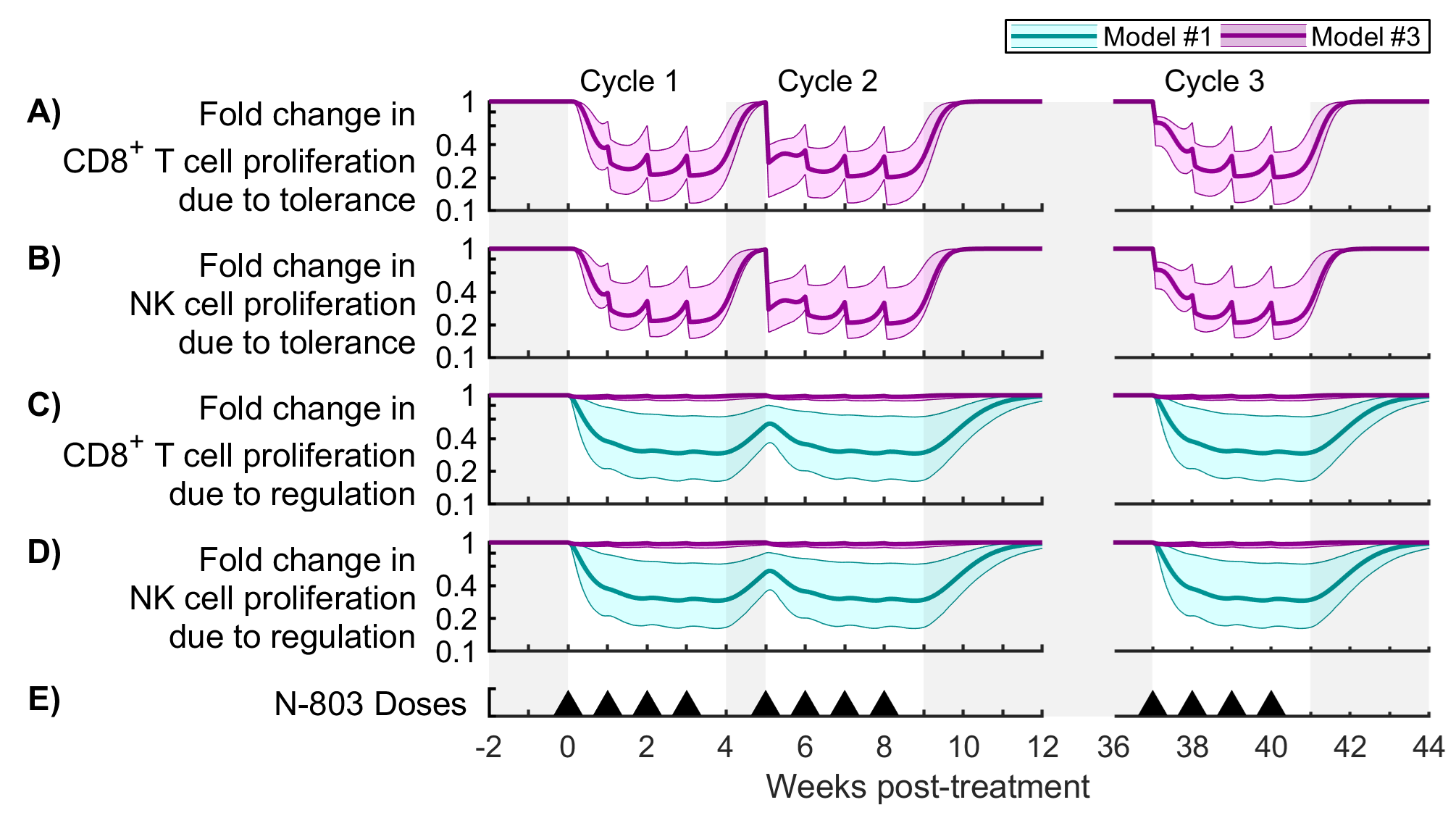

Supplement: S3 Fig — Shown are measures of mechanism contribution to CD8+ T cell and NK cell proliferation for the model with immune regulation and viral escape (cyan model #1) and the model with immune regulation and drug tolerance (purple model #3). Panels (A,B) show the fold change in CD8+ T cell proliferation and NK cell proliferation due to tolerance (Eq 30). Panels (C,D) show the fold change in CD8+ T cell proliferation and NK cell proliferation due to regulation (Eq 29). The bold line corresponds to the best-fit model, and the shaded region corresponds to the Bayesian 95% credible interval. See S1 Fig for corresponding parameter distributions. Panel (E) shows timing of 0.1 mg/kg subcutaneous doses of N-803. (TIF) [file pcbi.1009204.s003.tif]

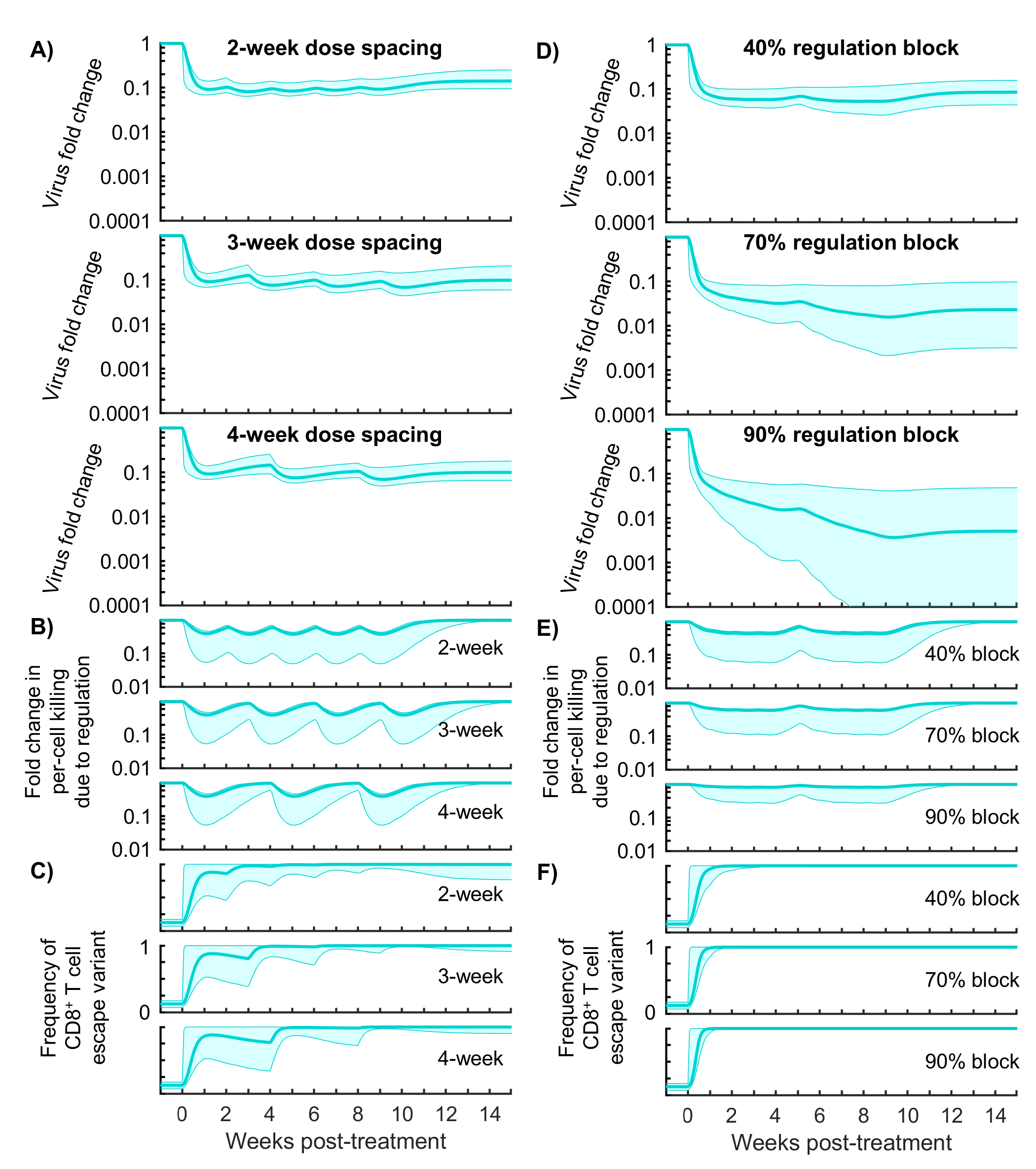

Supplement: S4 Fig — Panels (A-C) show the results of changing the N-803 dosing frequency for the model with immune regulation and viral escape (model #1). Panel (A) shows the fold change in viral load corresponding to the 0.1 mg/kg subcutaneous dosing regimens with 2-, 3-, and 4-week dosing. Panel (B) shows the corresponding fold changes in per-cell killing due to regulation (Eq 25). Panel (C) shows the corresponding changes in the frequency of the CD8+ T cell escape variant. Panels (D-F) show the response of model #1 to the 1-week dosing regimen (Fig 2) delivered along with regulatory blockade (simulated by 40, 70, and 90% reduction of killing regulation parameter λ). The bold line corresponds to the best-fit model, and the shaded region corresponds to the Bayesian 95% credible interval. (TIF) [file pcbi.1009204.s004.tif]

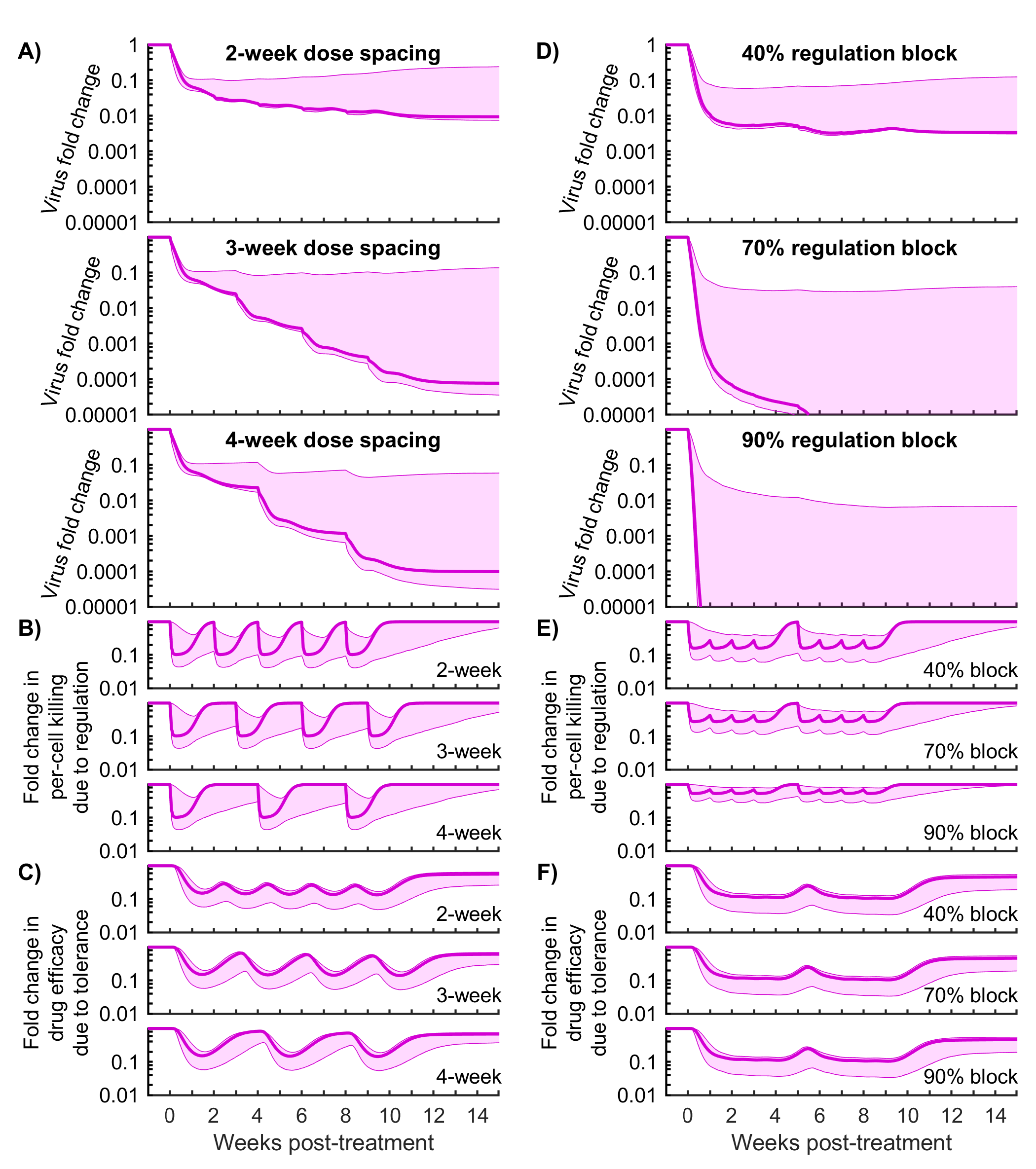

Supplement: S5 Fig — Panels (A-C) show the results of changing the N-803 dosing frequency for the model with immune regulation and drug tolerance (model #3). Panel (A) shows the fold change in viral load corresponding to the 0.1 mg/kg subcutaneous dosing regimens with 2-, 3-, and 4-week dosing. Panel (B) shows the corresponding fold changes in per-cell killing due to regulation (Eq 25). Panel (C) shows the corresponding fold changes in drug efficacy due to tolerance (Eq 24). Panels (D-F) show the response of model #3 to the 1-week dosing regimen (Fig 2) delivered along with regulatory blockade (simulated by 40, 70, and 90% reduction of killing regulation parameter λ). The bold line corresponds to the best-fit model, and the shaded region corresponds to the Bayesian 95% credible interval. (TIF) [file pcbi.1009204.s005.tif]
