# Supplementary material for: Mathematical modeling of N-803 treatment in SIV-infected non-human primates
Source: PLoS Comput Biol. 2021 Jul 28;17(7):e1009204. doi: 10.1371/journal.pcbi.1009204 (PMC8351941; doi:10.1371/journal.pcbi.1009204)
Supplement: S1 Appendix — (DOCX) [file pcbi.1009204.s006.docx]

**Mathematical modeling of N-803 treatment in SIV-infected non-human primates: S1 Appendix**

[Supplemental Methods 2](#_Toc70338897)

[Model assumptions 2](#_Toc70338898)

[Parameter space 3](#_Toc70338899)

[Parameter estimation algorithm 4](#_Toc70338900)

[Model comparison criteria 4](#_Toc70338901)

[Per-cell killing derivation 5](#_Toc70338902)

[Significance testing 6](#_Toc70338903)

[Sensitivity analysis 6](#_Toc70338904)

[Supplemental Results 8](#_Toc70338905)

[Discussion of sensitivity analysis 8](#_Toc70338906)

[Comparison to IL-15 receptors and inhibitory markers 10](#_Toc70338907)

[Model with long-term immune regulation 12](#_Toc70338908)

[Individual Fitting 14](#_Toc70338909)

[References 27](#_Toc70338910)

# Supplemental Methods

## Model assumptions

The following is a discussion of noteworthy assumptions in the model (Eq. 1-14 in the main text). Healthy target cells are assumed constant, which follows from two considerations. First, it was observed that total peripheral blood CD4^+^ T cells remained approximately constant during the N-803 treatment under consideration [1]. Second, during chronic HIV infection, only about ~2% of HIV-infected cells are replication-competent [2]. Taken together, these support the assumption that the healthy CD4^+^ T cells remained approximately constant over the time periods under consideration in this work.

A quasi-steady-state for virions relative to infected CD4^+^ T cells was assumed. Both HIV and SIV are cleared quickly from the plasma, with a virion half-life on the order of minutes [3-5]. Therefore, any delay between changes in infected cells and changes in virions was on a much shorter timescale than the dynamics of the observed system. The quasi-steady state implies that virions are approximately proportional to infected cells (i.e. 𝘝≈𝘬𝘐_𝘝_ and 𝘞≈𝘬𝘐_𝘞_) and allows model parameters to be calibrated to experimentally measured changes in viral load. The assumptions of constant target cells and quasi-steady-state of the free virus relative to infected cells have been used together in models of HIV treatment [6, 7].

**Cell types and activation**. We convolved all CD8^+^ T cells into one variable (𝐸), and we convolved all NK cells into another variable (𝐾). Killing rate constants (𝑔_𝐸_,𝑔_𝐾_) were applied to each of these total populations. Modifications to killing rate via drug stimulation, drug tolerance, and immune regulation (Eq. 7,8) represented changes in both the frequency of cytotoxically active cells within their respective total populations and changes in the individual efficacy cytotoxically active cells. We also neglect any delay between changes in antigen-dependent memory CD8^+^ T cell activation and changes in the overall killing rate, based on multiple considerations. First, chronic infections such as SIV include persistent CD8^+^ T cell activation. Second, memory CD8^+^ T cells acquire cytotoxic effector functions within 24 hours after antigen stimulation [8, 9]. Third, IL-15 promotes bystander activation of CD8^+^ T cells [10-12]. Such cells were capable of non-specific cytotoxicity of hepatitis-A-infected cells in a manner similar to NK cells [13].

**Immune regulation and drug tolerance**. We employ phenomenological representations of immune regulation and drug tolerance, with the two mechanisms being distinguished by their effect and their dynamics. Immune regulation directly reduces rates of killing and proliferation for CD8^+^ T cells and NK cells (via 𝜆,𝜑), while drug tolerance inhibits N-803 stimulation of these processes (via 𝜂). Both the generation and decay of the immune regulatory signal is governed by a single parameter (𝛿_REG_), while drug tolerance is governed by two parameters (𝛿_TOL_,𝜏). The tolerance recovery parameter (𝜏) allows drug tolerance to persist across long gaps in treatment, while immune regulation cannot persist long-term. We make no assumptions as to the sources of immune regulation or drug tolerance. For example, IL-15 receptor expression (modeled by drug tolerance) can be modulated by a variety of signals. CD122 expression is increased following antigen stimulation [14-16], while IL-6 can inhibit the upregulation of CD122 in follicular helper T cells [17]. IL-15 increased CD122 expression in memory CD8^+^ T cells of NHPs [18]. In our data, a transient increase in CD122 was also observed in effector memory CD8^+^ T cells during the first few weeks of treatment [1]. The effect of native cytokine signaling, including IL-15, is convolved into rate constants (e.g. 𝑔_𝐸_,𝑔_𝐾_,𝑑_𝐸_,𝑑_𝐾_). We also convolve the effect of receptor levels on native cytokine activity into drug stimulation, immune regulation, and drug tolerance terms (Eq. 7-8).

## Parameter space

The following is a discussion of the fixed parameters in Table 3. Initial conditions for SIV plasma viral load, CD8^+^ T cells, and NK cells, where the respective means of pre-treatment data across all 3 subjects (15 samples total for each species) [1]. Initial N-803 at the absorption site was based on measured N-803 molecular weight of 114 kDa [19] and the administered dose of 0.1 mg/kg [1]. The N-803 absorption rate constant (𝑘_𝑎_) was obtained from the elimination phase of the plasma pharmacokinetics following a 10 μg/kg subcutaneous dose in humans participating in cancer trials [20]. N-803 clearance rate constant (𝑘_𝑒_) was obtained from the half-life (7.97 ± 1.29 h) reported following a 0.1 mg/kg intravenous dose in cynomolgus macaques [21]. The ratio of the N-803 volume of distribution and bioavailability (𝑣_𝑑_/𝐹) was obtained from the volume of distribution (37.56 ± 9.1 mL/kg) reported for a 0.1 mg/kg intravenous dose in cynomolgus macaques [21] and the bioavailability (0.0299 ± 0.0160) reported for a 10 μg/kg subcutaneous dose in humans [20]. The number of tolerance variables (𝑁) and number of regulation variables (𝑀) were chosen to reflect dynamics of N-803 receptors CD122 and CD132 and inhibitory markers CD39 and PD-1, respectively, on CD8^+^ T cells and NK cells [1]. The number of tolerance variables (𝑁) was higher to reflect the delay in receptor changes with respect to inhibitory marker changes.

The following is a discussion of the fitted parameters in Table 3. The N-803 50% effect concentration (𝐶_50_) was based on the ex vivo 50% effect concentration for CD8^+^ T cells and NK cells in rhesus macaques (estimated as 10-1000 pM from figure) [22]. The lower limit was then adjusted to account for the possibility of higher concentrations of N-803 in the lymph tissue relative to the blood, as evidenced by murine tissue biodistribution data [21]. The CD8^+^ T cell killing rate constant (𝑔_𝐸_) was based on the range of estimates of total HIV-infected cell death rate due to CD8^+^ T cells (reviewed in [23]) and peripheral blood concentration of ~500 CD8^+^ T cells per μL (used as initial condition). NK cell killing rate constant (𝑔_𝐾_) was assumed to be some fraction of CD8^+^ T cell killing rate based on comparison of viral load after CD8 depletion (elimination of CD8^+^ T cells and NK cells) [24] and CD16 depletion (elimination of NK cell cytotoxic subgroup) [25]. The cell death rate constants (𝑑_𝐸_, 𝑑_𝐾_) were based on CD8^+^ T cell and NK cell turnover in SIV-infected rhesus macaques [26]. The upper limits were increased to fit the rate of contraction following N-803 observed in the NHP data [1]. Thus, the value incorporates changes in survival signals due to N-803 treatment and immune regulation. Proliferation stimulation factors (𝜌_𝐸_, 𝜌_𝐾_) were limited according to maximum allowed expansion rates (𝜌_𝐸_·𝑑_𝐸_, 𝜌_𝐾_·𝑑_𝐾_). These rates were derived from Eq. (3,4,8) by assuming 𝘩 >> [𝐸],[𝐾]. The maximum expansion rates are limited based on CD8^+^ T cell clonal expansion rate for rhesus macaques (~1/day) [27].

## Parameter estimation algorithm

We calibrated the model using a multi-start local search approach implemented in MATLAB version R2018b (Mathworks). The parameter space (Table 3) was sampled on a logarithmic scale via Latin hypercube sampling [28] using the MATLAB ‘lhsdesign’ function (10,000 samples). Each sample set of parameter values was used as an initial guess in an interior-point optimization algorithm [29] implemented by the MATLAB ‘fmincon’ function. This algorithm, also operating on the logarithmic parameter values, returned a local minimum of the negative loglikelihood (Eq. 20) with respect to the log-fold change in virus, fold change in CD8^+^ T cells, and fold change in NK cells in all three subjects. Some viral data points lay on the lower limit of detection for the viral assay and were omitted from the likelihood function. Parameters sets associated with the highest likelihood were used to instantiate a Markov Chain Monte Carlo algorithm to generate a sample of parameter values from posterior distributions.

## Model comparison criteria

We also considered qualitative observations of the viral data that should be present in a suitable model, which were quantified as follows. First, there was a viral rebound in treatment cycle 1 (Fig 4I). This was represented by the difference in the viral load at the end of cycle 1 (week 4) and the minimum viral load in week 4 (Eq. S1). In these equations, 𝑉 stands for total virus (𝑉+𝑊).

$$\text{Criteria (I)}=\log V(4)-\mathrm{logmin}(V(0:4))$$

Second, the viral response in cycle 2 was weaker than that in cycle 3 (Fig 4J). This was quantified by the difference in viral load drops between the two cycles (Eq. S2).

$$\text{Criteria (J)}=[\log V(37)-\mathrm{logmin}(V(37:41))]-[\log V(5)-\mathrm{logmin}(V(5:9))]$$

Third, the response in cycle 3 was weaker than that in cycle 1 (Fig 4K). This was defined as above (Eq. S3).

$$\text{Criteria (J)}=[\log V(0)-\mathrm{logmin}(V(0:4))]-[\log V(37)-\mathrm{logmin}(V(37:41))]$$

## Per-cell killing derivation

In order to quantify the effect of immune regulation, drug tolerance, and viral escape on per-cell cytotoxic activity, we defined per-cell killing (PCK). The following is a derivation of the expression for per-cell killing (PCK). To begin, the rates of change for each viral variant can be added together to describe the rate of change of the total virus (Eq. S4).

$$\left[V\right]^{'}+\left[W\right]^{'}=q_{V}\left[V\right]+q_{W}\left[W\right]-g_{E}\left[E\right]\left(\left[V\right]+\chi\left[W\right]\right)-g_{K}\left[K\right]\left(\left[V\right]+\left[W\right]\right)$$

We next introduce *v*, *w*, *e*, *k* as frequencies of virus or killer cells within their respective groups (e.g. [𝑣]=[𝑉]/([𝑉]+[𝑊]) or [𝑒]=[𝐸]/([𝐸]+[𝐾])). Collecting terms in Eq. (S5) results in an expression that applies to the sum of CD8^+^ T cells and NK cells and the sum of both viral variants .

$$\left[V+W\right]^{'}=\left(q_{V}\left[v\right]+q_{W}\left[w\right]\right)\left[V+W\right]-\left\{\left(\left[v\right]+\chi\left[w\right]\right)g_{E}\left[e\right]-g_{K}\left[k\right]\right\}\left[E+K\right]\left[V+W\right]$$

There is a collection of terms that behaves the same way as killing rate constants (𝑔_𝐸_,𝑔_𝐾_) in Eq. (1,2). This expression is the per-cell killing (PCK) absent N-803 intervention (Eq. S6).

$$\text{PCK}=\frac{[\text{total killing rate}]}{[\text{killer cells}][\text{virus}]}=\left(\left[v\right]+\chi\left[w\right]\right)g_{E}\left[e\right]-g_{K}\left[k\right]$$

Supplementing this with changes in killing rate due to N-803 stimulation, drug tolerance, and immune regulation (Eq. 7 in main text) results in the expression for PCK during N-803 treatment (Eq. S7-S9 or Eq. 22-24 in the main text).

## Significance testing

Statistical comparison of the quality criteria between models (Fig 5I-K) was done in MATLAB version R2018b (Mathworks) using the Tukey test. Statistical comparison of the results of treatment exploration (Fig 8) was conducted in GraphPad Prism 8. Separate analyses were conducted for changes due to dose spacing (Fig 8A) and for changes due to regulation blockade (Fig 8B), but the same technique was used. For example, this was a two-way ANOVA incorporating the model (#1 vs #3) and the dose spacing (2-4 weeks). Data points were matched by model parameter set, employing the Geisser-Greenhouse correction for non-sphericity. Thus, we treated the results from each parameter as though they had come from the same subject in a longitudinal study. The Tukey test for multiple comparisons was conducted to test the statistical significance of the difference of means between each dose spacing. In addition, one-sample t-test was used to assess if each result was different than zero (zero being the case where there was no improvement with respect to the control regimen).

## Sensitivity analysis

A sensitivity analysis was conducted to quantify the correlations between model parameters and the treatment efficacy over multiple timescales in the full model. As a measure of treatment efficacy, we considered the drop in viral load for each treatment cycle (Fig S6). This was defined as the difference between the viral load at the start of the cycle (e.g. viral load at week 0) and the minimum viral load across that cycle (e.g. minimum viral load between week 0 and week 4). We used partial rank correlation coefficients (PRCC) calculated via the MATLAB ‘partialcorr’ function [30]. A Latin hypercube sample of 10,000 parameter sets was generated from a wide parameter space (Table S1). The model was evaluated at each parameter set, and PRCC were calculated between each parameter and the viral load drop for each of the three treatment cycles. Correlations that were significant at α=0.0001 across three repetitions of 10,000 samples were considered valid.


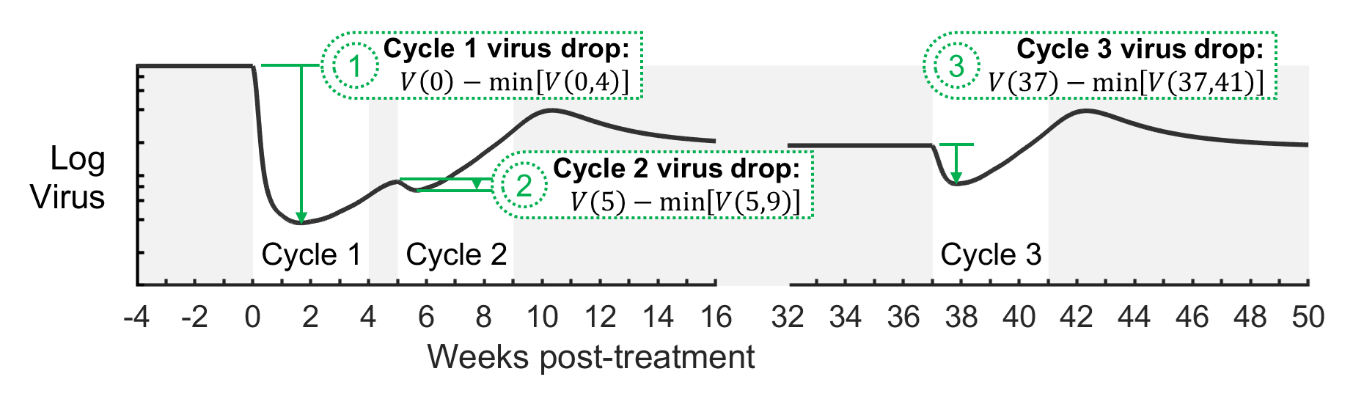


**Fig S6. Metrics for N-803 treatment efficacy considered during sensitivity analysis.** The drop in viral load during each treatment cycle (1,2,3) was used as a measure of treatment efficacy. The viral load drop is defined as the difference between the viral load at the start of the cycle (e.g. viral load at week 0) and the minimum viral load across that cycle (e.g. minimum viral load between week 0 and week 4). Each metric is highlighted using a representative viral trajectory.

**Table S1. Model parameters varied during sensitivity analysis.**

| **Parameter** | **Symbol** | **Range** | **Units** |
| --- | --- | --- | --- |
| N-803 50% effect concentration | 𝐶_50_ | (0.4, 40) | pM |
| Tolerance rate constant | 𝛿_TOL_ | (0.05, 5) | /day |
| Regulation rate constant | 𝛿_REG_ | (0.05, 5) | /day |
| Tolerance recovery | 𝜏 | (0.001,1) |  |
| Escape variant initial frequency | 𝑓 | (0.001, 1) |  |
| Escape variant susceptibility factor | 𝜒 | (0.001, 1) |  |
| CD8^+^ T cell death rate constant | 𝑑_𝐸_ | (0.01, 1) | /day |
| NK cell death rate constant | 𝑑_𝐾_ | (0.01, 1) | /day |
| CD8^+^ T cell killing rate constant | 𝑔_𝐸_ | (10^-5^, 0.01) | μL/#·d |
| NK cell killing rate constant | 𝑔_𝐾_ | (10^-5^, 0.01) | μL/#·d |
| Maximum proliferating cells | 𝘩 | (50, 5000) | #/μL |
| CD8^+^ T cell proliferation stimulation factor | 𝜌_𝐸_ | (0.1, 10) |  |
| NK cell proliferation stimulation factor | 𝜌_𝐾_ | (0.1, 10) |  |
| CD8^+^ T cell killing stimulation factor | 𝛾_𝐸_ | (0.01, 100) |  |
| NK cell killing stimulation factor | 𝛾_𝐾_ | (0.01, 100) |  |
| Tolerance effect factor | 𝜂 | (0.01, 100) |  |
| Proliferation regulation factor | 𝜑 | (0.1, 10) |  |
| Killing regulation factor | 𝜆 | (0.01, 100) |  |

Shown are the allowed ranges for parameter values during sensitivity analysis. Parameters were sampled logarithmically from the given ranges via Latin hypercube sampling [28]. Parameters not shown were fixed (Table 3).

# Supplemental Results

## Discussion of sensitivity analysis

To assess the relative impact of each treatment response mechanism on viral responses, we performed global sensitivity analysis. This analysis correlates changes in parameter values to changes in model outputs in the context of other parameter influences. We calculated the partial rank correlations coefficients (PRCCs) (Table S2) between model parameters and model outputs of interest. The parameters of interest are those governing drug tolerance, immune regulation, and viral escape. The outputs of interest are the viral load drop during each treatment cycle which we use as a metric of treatment efficacy in each cycle (Fig S6).

Parameters governing immune regulation had strong correlations with treatment efficacy. Strong killing regulation (i.e. high 𝜆) is associated with low efficacy (small viral drop) in all three cycles, having the strongest impact in cycle 1. Strong proliferation regulation (i.e. high 𝜑) correlates with lower efficacy (smaller viral drop) in cycle 2, reflecting the delay that comes from acting on viral load indirectly through suppressing CD8^+^ T cell and NK cell population expansion. A fast regulatory response (i.e. high 𝛿_REG_) correlated with lower efficacy (smaller viral drop) in cycle 1 and cycle 3. In contrast, fast regulatory response was also associated with higher efficacy in cycle 2, which follows a shorter 2-week break in treatment. Recall that high 𝛿_REG_ also causes regulation to abate quickly after treatment. Taken together, these correlations indicate that: 1) successive doses are more effective if they are timed such that the regulatory signal is allowed to abate between doses; and 2) directly blocking regulation (e.g. lowering 𝜆 or 𝜑) could improve treatment response to N-803.

Correlations between tolerance parameters and treatment efficacy mirrored those of regulation, with some key differences. Tolerance strength (𝜂) had correlations that fell between those of killing regulation strength (𝜆) and proliferation regulation strength (𝜑). This reflects how tolerance strength (𝜂) reduces N-803 stimulation of both cytotoxicity and proliferation of CD8^+^ T cells and NK cells. Rapid onset of tolerance (i.e. high 𝛿_TOL_) was also correlated with lower treatment efficacy in cycle 1. Unlike regulation speed (𝛿_REG_), the direction of the correlation for tolerance speed did not change for cycle 2. Based on the NHP data, the drug tolerance model mechanism was structurally slower than regulation based on the number of delay variables (𝑁 = 6 for tolerance and 𝑀 = 2 for regulation). With tolerance being slower to adapt, the 2-week break between cycles 1 and 2 was too short to allow the effects of tolerance to subside. However, the tolerance recovery parameter (𝜏) had a comparably positive correlation to the treatment effectiveness in cycle 3 as regulation speed had to cycle 2. Together, this reflects how tolerance and regulation effects of previous treatment cycles may alter the outcome of the subsequent treatment cycles.

High initial frequency of the escape variant (i.e. high 𝑓) is correlated with lower treatment efficacy in cycle 1 but not cycle 2. This difference between cycles 1 and 2 is because the escape variant (𝑊) largely replaced the dominant variant (𝑉) due to the selective pressure of treatment in cycle 1. The correlation reappears in cycle 3, as the original dominant variant recovers in the break between cycles 2 and 3 due to a fitness advantage over the escape variant. High susceptibility of the escape variant to CD8^+^ T cells (i.e. high 𝜒) was associated with higher treatment efficacy in all three treatment cycles. This consistent correlation across cycles is indicative of how viral escape, as modeled here, leads to persistent loss of efficacy.

**Table S2. Sensitivity analysis.**

| **Measure of treatment efficacy** | **Killing**  **Regulation**  **Strength** | **Proliferation**  **Regulation**  **Strength** | **Regulation**  **Speed** | **Tolerance**  **Strength** | **Tolerance**  **Speed** | **Tolerance**  **Recovery** | **Variant W**  **Initial**  **Frequency** | **Variant W**  **Susceptibility**  **to T cells** | **Dummy**  **Variable** |
| --- | --- | --- | --- | --- | --- | --- | --- | --- | --- |
|  | ***λ*** | ***ϕ*** | **δ_REG_** | ***Η*** | **δ_TOL_** | **τ** | ***f*** | **χ** |  |
| Cycle 1 virus drop | **−0.60** | **−0.13** | **−0.50** | **−0.21** | **−0.23** | - | **−0.30** | **+0.25** | - |
| Cycle 2 virus drop | **−0.49** | **−0.36** | **+0.07** | **−0.47** | **−0.07** | - | - | **+0.13** | - |
| Cycle 3 virus drop | **−0.35** | **−0.08** | **−0.39** | **−0.17** | **−0.07** | **+0.07** | **−0.06** | **+0.23** | - |

Shown are the partial rank correlation coefficients (PRCC) of select model parameters to N-803 treatment efficacy, as measured by the drop in viral load during each cycle (Fig S6). The strongest possible negative correlation is **−**1, and the strongest possible positive correlation is +1. Correlations shown had p-value <= 0.00001 across three repetitions of 10,000 samples

## Comparison to IL-15 receptors and inhibitory markers

Additional data collected along with our training data validated the timing of immune regulation and drug tolerance. Specifically, the immune regulation REG_M_ (Eq. 7-8,14) increased shortly after treatment began and decayed shortly after treatment ended (Figs S7A,B). This agrees well with markers indicative of immune regulation, such as inhibitory marker expression on CD4^+^ T cells, CD8^+^ T cells, and NK cells (Figs S7C-E). The speed of regulation depended on the model. When drug tolerance was present (model #3) immune regulation was potentially very fast. In contrast, drug tolerance had a slower onset (Fig S7F), shown as TOL_N-1_ + TOL_N_ (Eq. 7-8, 11-12). Tolerance was not substantial until the second week, which slightly precedes the observed decline in N-803 receptors observed in the data (Fig G-I). In summary, the timing of modeled immune regulation and drug tolerance was consistent with markers of the mechanisms they represent.


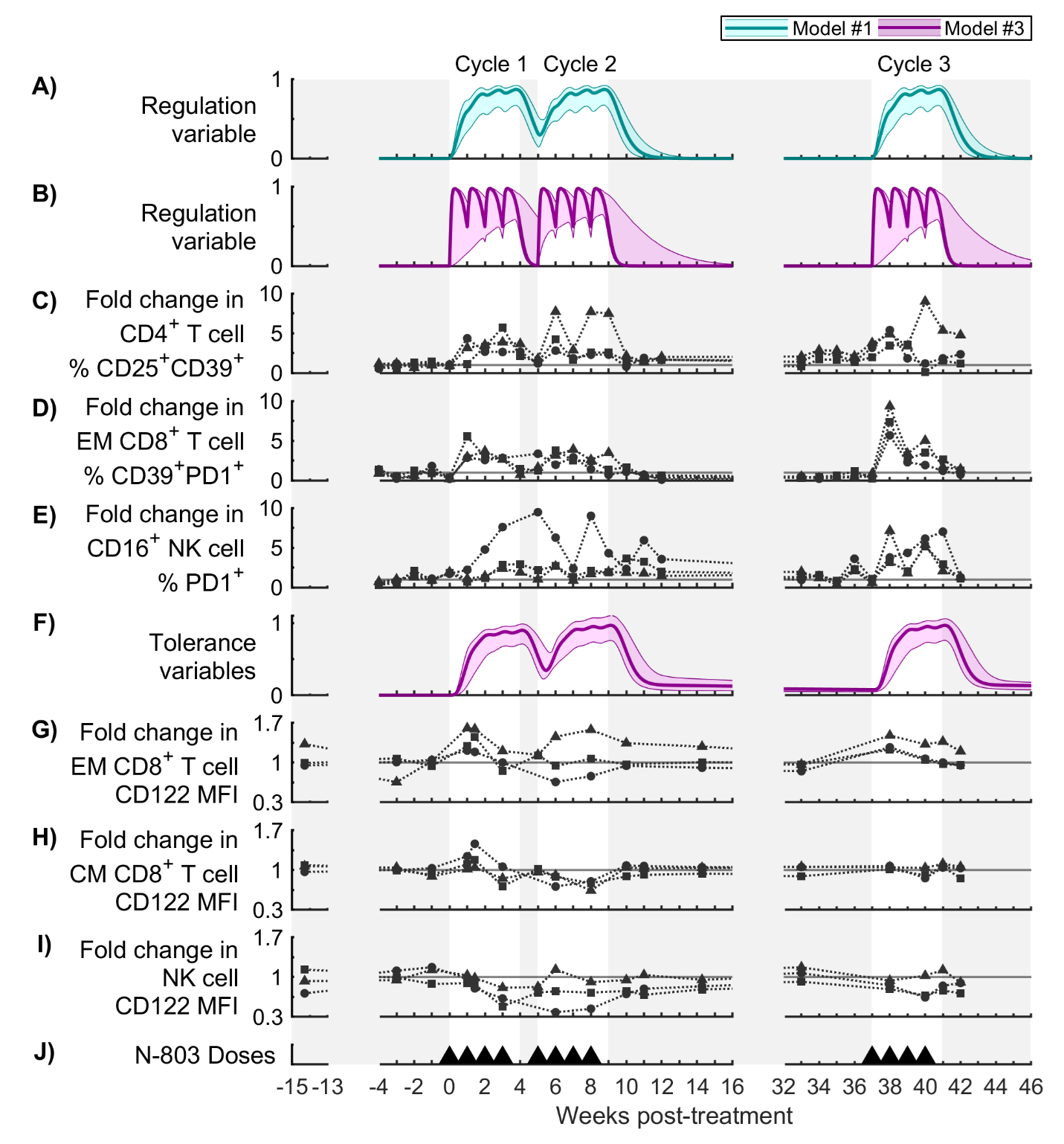


**Fig S7. Comparison of regulation dynamics in model and data.** Panels (A,B) show immune regulation REG_M_ (Eq. 7-8,14) in model #1 and model #3, respectively. Panel (F) shows drug tolerance TOL_N-1_ + TOL_N_ (Eq. 7-8, 11-12) in model #3. The bold line corresponds to the best-fit model, and the shaded region corresponds to the Bayesian 95% credible interval. Panels (C-E) and (G-H) show changes in expression of inhibitory markers and IL-15 receptor subunits during N-803 treatment (selected from [1]). Panel (C) shows changes in the frequency of CD25^+^CD39^+^ cells among CD4^+^ T cells (i.e. regulatory T cells). Panel (D) shows changes in the frequency of CD39^+^PD1^+^ cells among effector memory CD8^+^ T cells. Panel (E) shows changes in the frequency of PD1^+^ cells among CD16^+^ NK cells. Panels (G-I) show changes in the expression of the IL-15 receptor subunit (CD122) on effector memory CD8^+^ T cells, central memory CD8^+^ T cells, and NK cells. For each NHP, data is normalized to the mean of pre-treatment data points.

## Model with long-term immune regulation

It is conceivable that N-803 induced deviations in immune regulatory signals could persist across the long treatment gap, which could provide a simpler explanation of long-term CD8^+^ T cell, NK cell, and SIV dynamics. To this end, a model with long-term regulation, and no drug tolerance, was also calibrated to the NHP data. This model deviates from the full model (Eq. 1-18) by replacing Eq. (7,8) with Eq. (S10,S11).

$$\begin{matrix} g_{i}\to g_{i}\left[1+\overbrace{\gamma_{i}\left(\frac{\left[C\right]}{C_{50}+\left[C\right]}\right)}^{\text{Drug Effect}}\right]\overbrace{\left(\frac{1}{1+\lambda\left([\text{TOL}_{N-1}]+[\text{TOL}_{N}]\right)}\right)}^{\text{Immune Regulation}} & i=E,K \end{matrix}$$

While the model was able to reproduce the dynamics of CD8^+^ T cells and NK cells (Fig S8B and S8C), the dynamics of SIV was poorly represented (Fig S8A). The viremia decayed to a setpoint during treatment cycle 1 without the subsequent rebound observed in the NHP data. Thus, allowing immune regulation to persist long-term, absent drug tolerance, does not qualitatively match the viral dynamics.


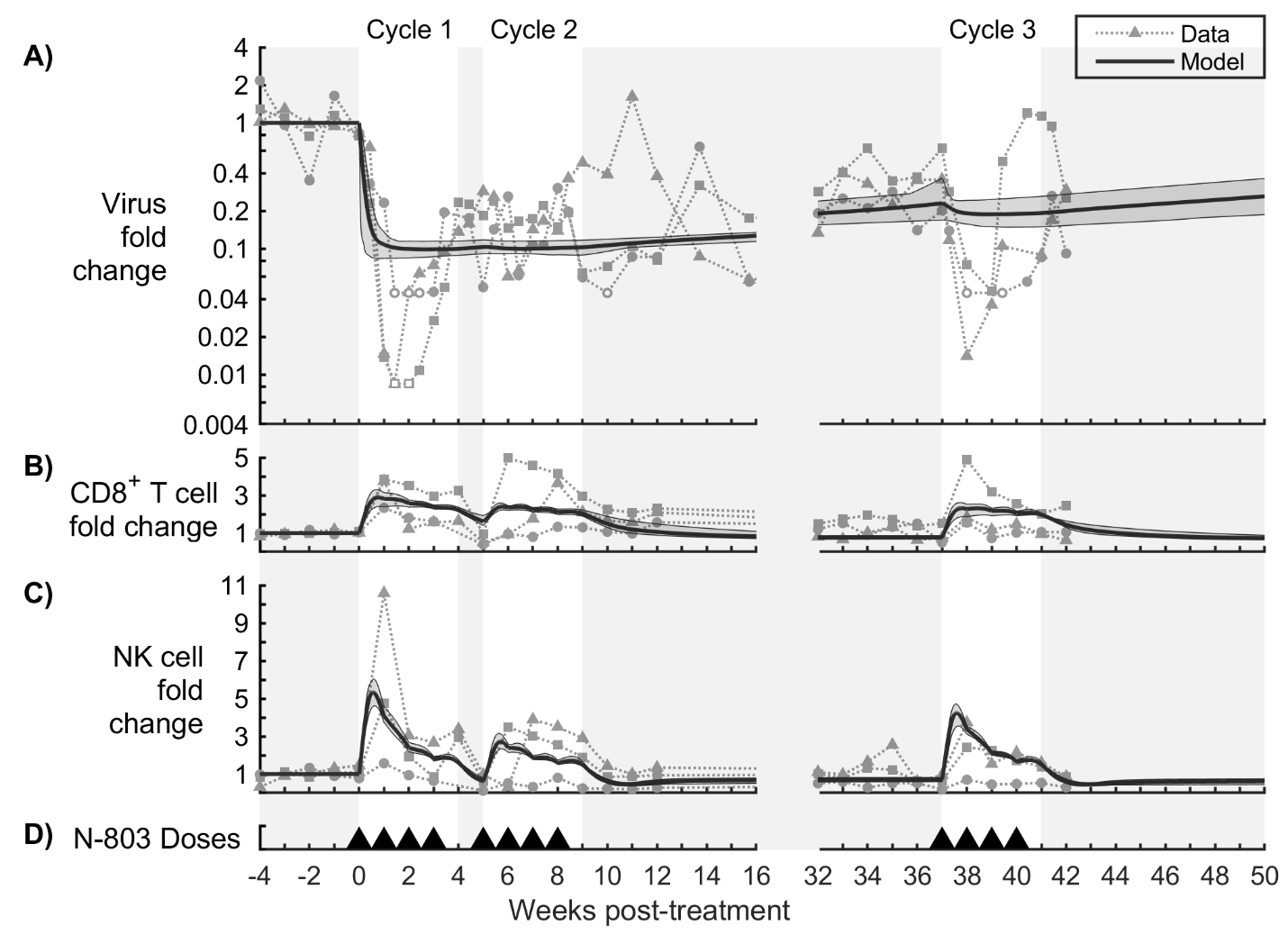


**Fig S8. Long-term regulation model calibration.** A model with long-term regulation, and no drug tolerance (see Eq. S10,S11), was calibrated to (A) fold change in virus in the plasma, (B) fold change in CD8^+^ T cells in the peripheral blood, and (C) fold change in NK cells in the peripheral blood. The bold line corresponds to the best-fit model, and the shaded region corresponds to the Bayesian 95% credible interval. Data from N-803-treated SIV-infected NHPs are shown as different symbols for each NHP [1]. Open symbols were at the lower limit of detection for the viral assay (100 CEQ/mL) and were omitted from parameter estimation. Panel (D) shows timing of 0.1 mg/kg subcutaneous doses of N-803.

## Individual fitting

Parameter estimation, uncertainty quantification, and model comparison were repeated utilizing data from each of the three subjects individually. Figures S9-S11 show the full model results (comparable to Fig 3 in the main text). Figures S12-S14 shows the model comparisons with respect to viral load (comparable to Fig 4). Figures S15-S17 show the model comparisons with respect to cytotoxic cells (comparable to Fig 5). Figures S18-S20 show the parameter distributions (comparable to Fig S1).

The inspection of individual fits lend support to the decision to fit all three subjects simultaneously. Some extreme model behaviors resulted from fitting to what could potentially be measurement noise. For example, The NK cells for subject r08016 showed a nearly 17-fold increase (Fig S9), driven chiefly by a single data point. Still, the results of model comparison held for subject r08016 and r09089 (Fig S12,S13). In short, model #1 (immune regulation and viral escape) and model #3 (immune regulation and drug tolerance) were the best models. These models had both low AICc and met all three quality criteria. Subject r11021, however, had viral load that was closer to the limit of detection of the essay. Thus, much of the cycle 1 decline and rebound observed in the other two subjects was censored in subject r11021. This resulted in all models having comparable fits to this subject’s data (Fig S14), owing to the simpler dynamics presented.


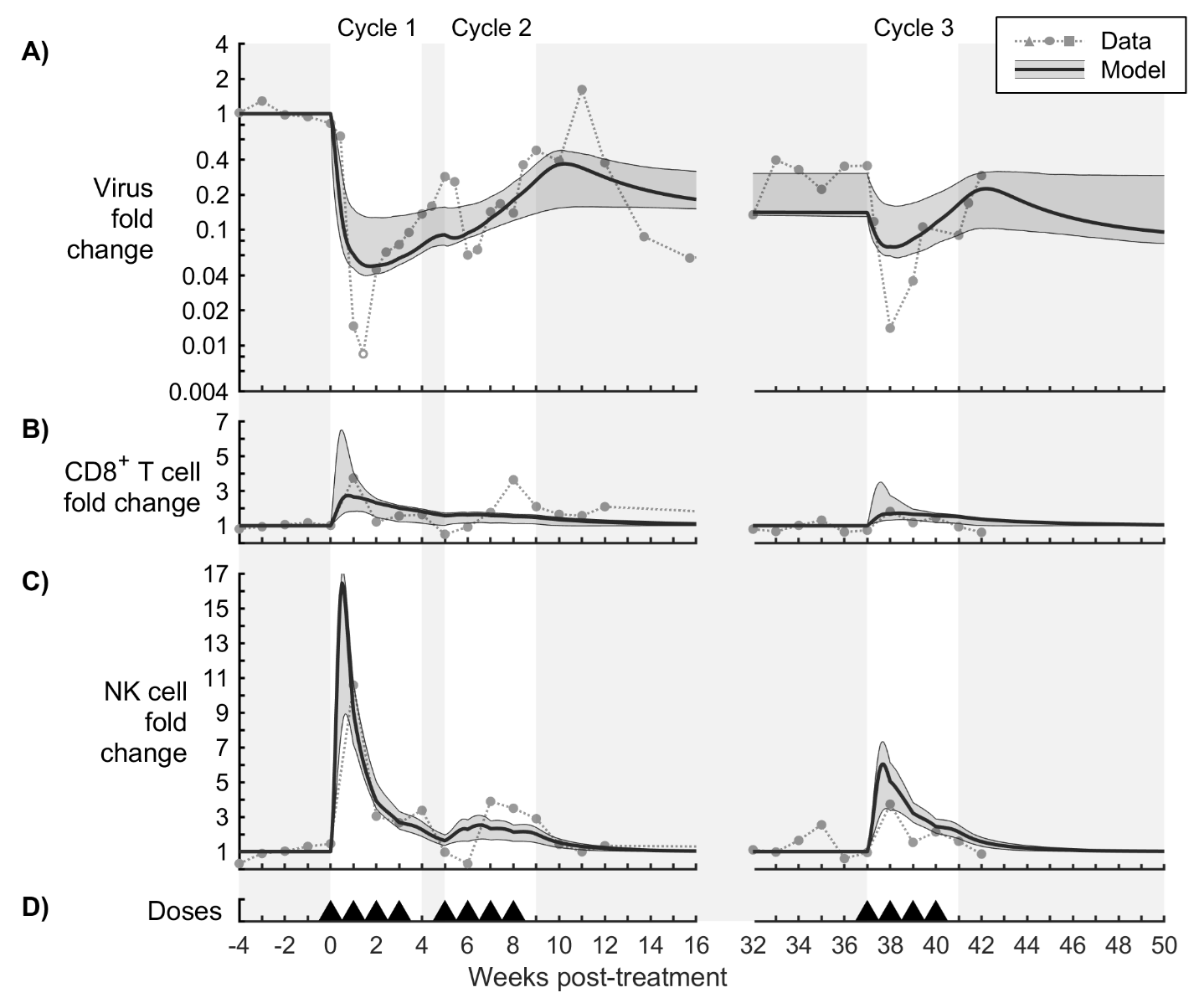


**Fig S9. Model calibration to N-803-treated SIV-infected NHP data (Subject r08016).** The model was calibrated to (A) fold change in virus in the plasma, (B) fold change in CD8^+^ T cells in the peripheral blood, and (C) fold change in NK cells in the peripheral blood. The bold line corresponds to the best-fit model, and the shaded region corresponds to the Bayesian 95% credible interval. See Figure S18 for corresponding parameter distributions. Data from the N-803-treated SIV-infected NHP is also shown [1]. Open symbols were at the lower limit of detection for the viral assay (100 CEQ/mL) and were omitted from parameter estimation. Panel (D) shows timing of 0.1 mg/kg subcutaneous doses of N-803.


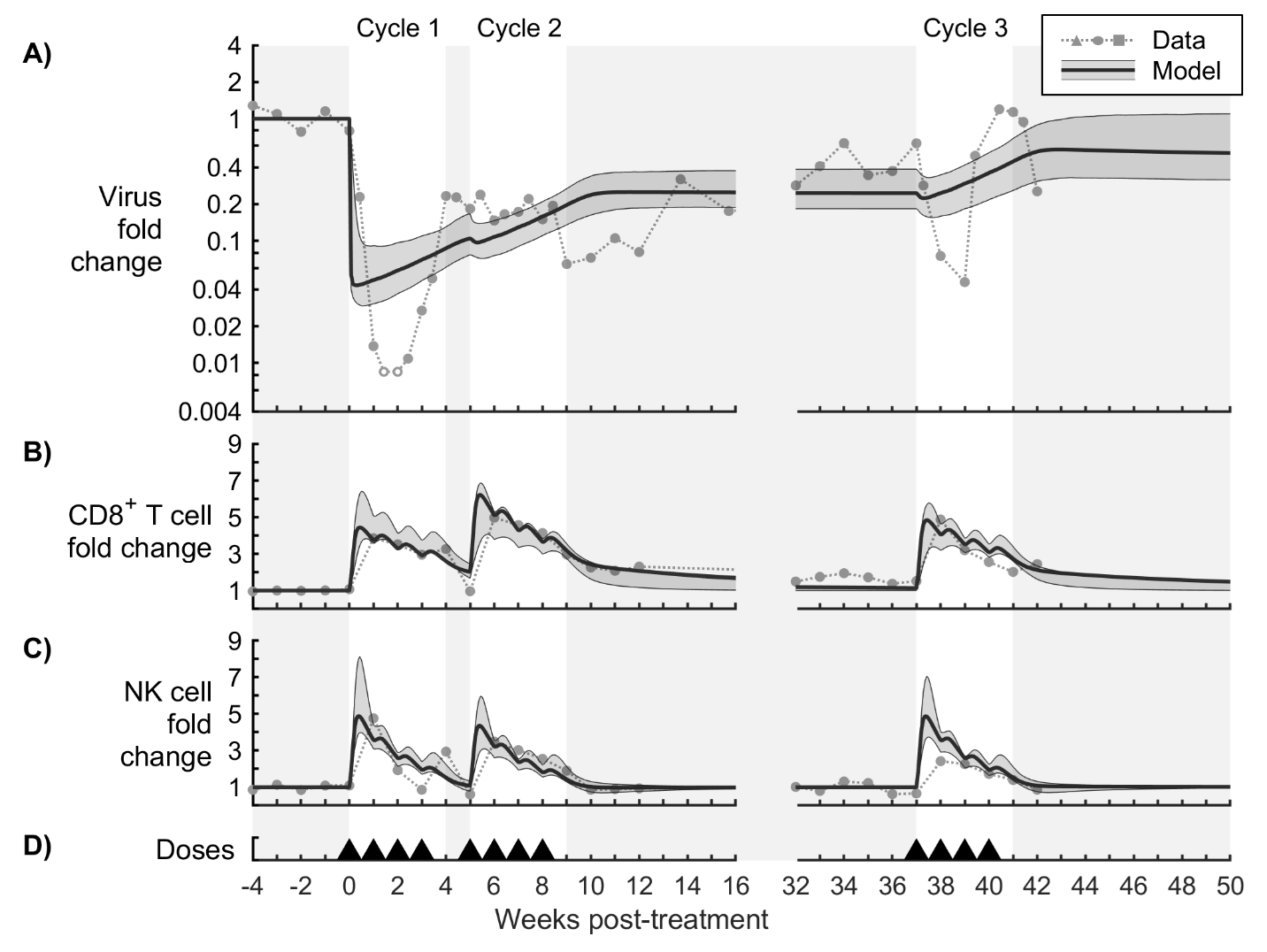


**Fig S10. Model calibration to N-803-treated SIV-infected NHP data (Subject r09089).** The model was calibrated to (A) fold change in virus in the plasma, (B) fold change in CD8^+^ T cells in the peripheral blood, and (C) fold change in NK cells in the peripheral blood. The bold line corresponds to the best-fit model, and the shaded region corresponds to the Bayesian 95% credible interval. See Figure S19 for corresponding parameter distributions. Data from the N-803-treated SIV-infected NHP is also shown [1]. Open symbols were at the lower limit of detection for the viral assay (100 CEQ/mL) and were omitted from parameter estimation. Panel (D) shows timing of 0.1 mg/kg subcutaneous doses of N-803.


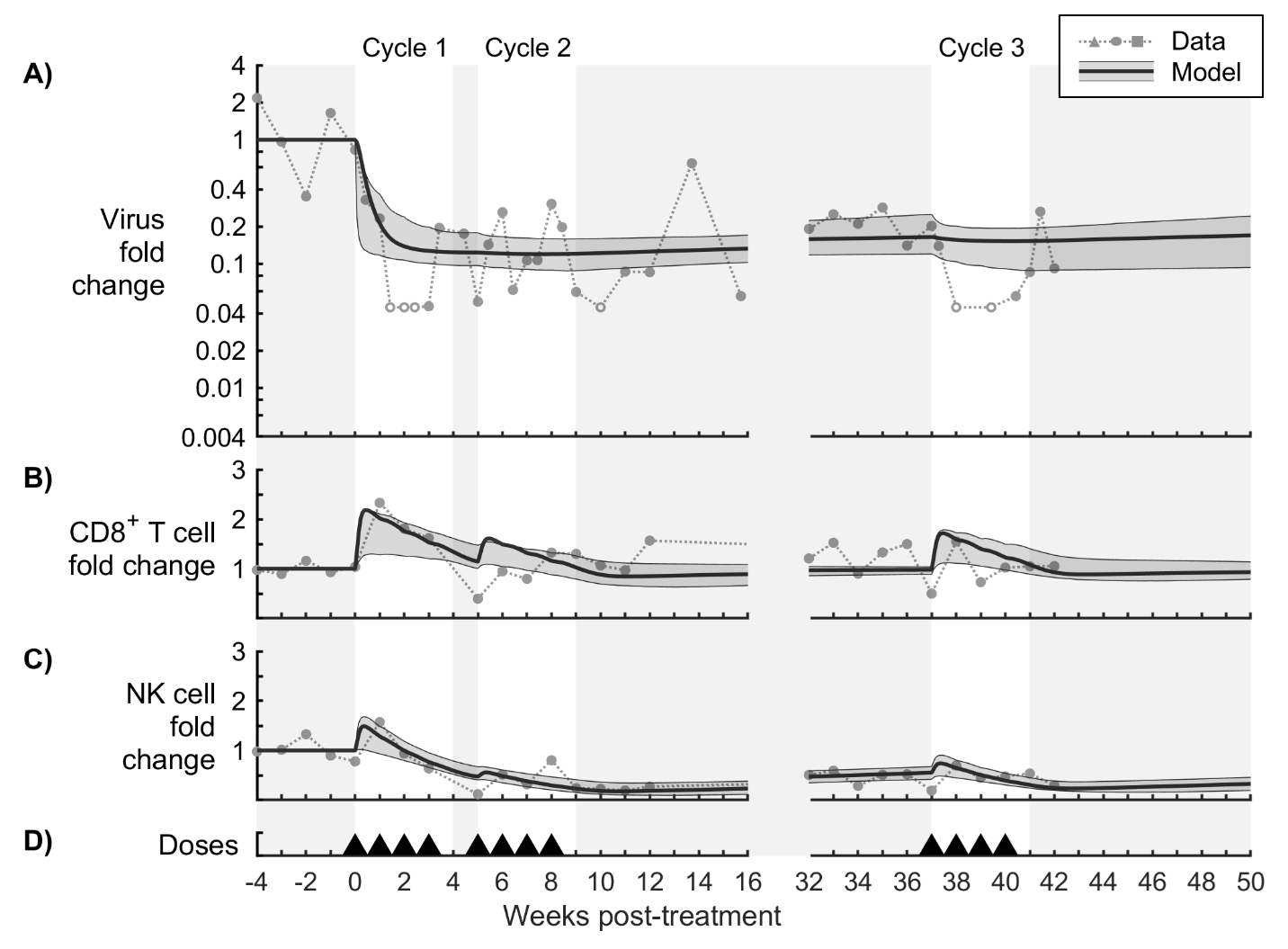


**Fig S11. Model calibration to N-803-treated SIV-infected NHP data (Subject r11021).** The model was calibrated to (A) fold change in virus in the plasma, (B) fold change in CD8^+^ T cells in the peripheral blood, and (C) fold change in NK cells in the peripheral blood. The bold line corresponds to the best-fit model, and the shaded region corresponds to the Bayesian 95% credible interval. See Figure S20 for corresponding parameter distributions. Data from the N-803-treated SIV-infected NHP is also shown [1]. Open symbols were at the lower limit of detection for the viral assay (100 CEQ/mL) and were omitted from parameter estimation. Panel (D) shows timing of 0.1 mg/kg subcutaneous doses of N-803.


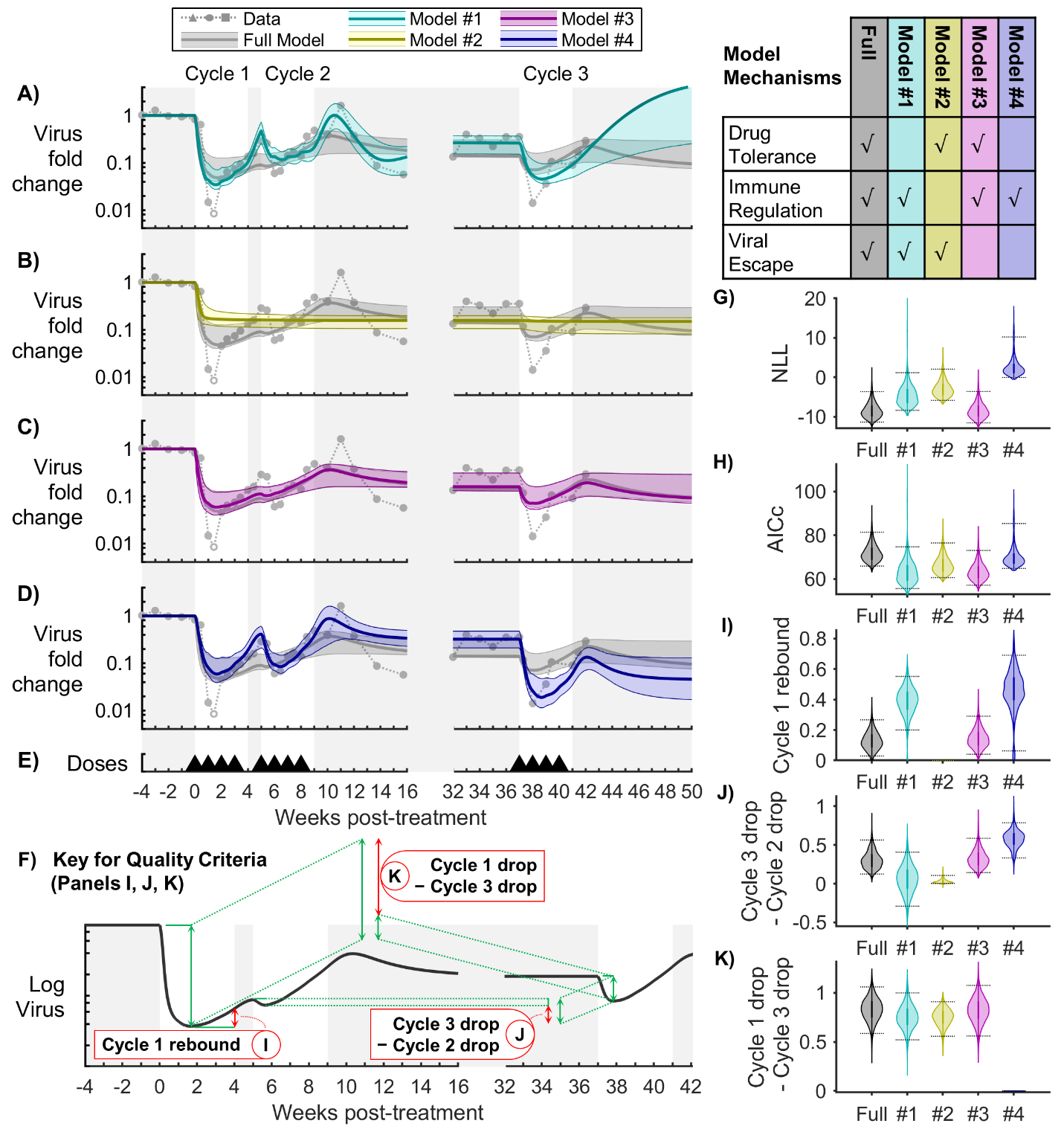


**Fig S12. Model comparison for viral load (Subject r08016).** Models with different combinations of mechanisms were compared to assess the importance of drug tolerance, immune regulation, and viral escape. Panels (A-D) compare the fold change in virus between the full model and models #1-4, respectively. The bold line corresponds to the best-fit model, and the shaded region corresponds to the Bayesian 95% credible interval. See Figure S18 for corresponding parameter distributions. Panel (E) shows timing of 0.1 mg/kg subcutaneous doses of N-803. Panels (G,H) show the corresponding Negative Log-Likelihood (NLL, Eq. 20) and Akaike Information Criterion (AICc, Eq. 21) for the Bayesian MCMC samples. Panels (I-K) show the three quality criteria, which are described in panel (F). Bayesian 95% credible intervals are marked.


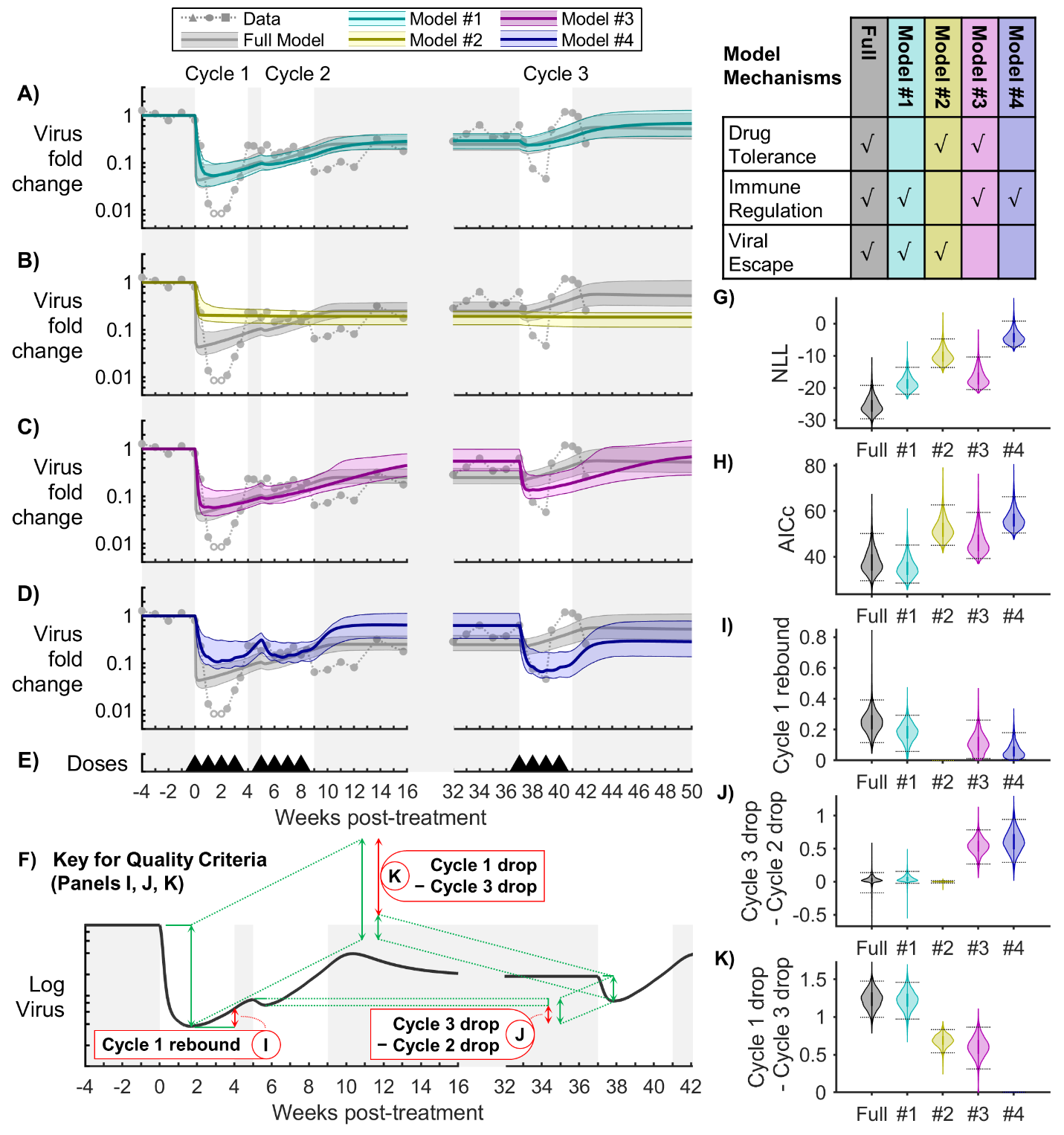


**Fig S13. Model comparison for viral load (Subject r09089).** Models with different combinations of mechanisms were compared to assess the importance of drug tolerance, immune regulation, and viral escape. Panels (A-D) compare the fold change in virus between the full model and models #1-4, respectively. The bold line corresponds to the best-fit model, and the shaded region corresponds to the Bayesian 95% credible interval. See Figure S19 for corresponding parameter distributions. Panel (E) shows timing of 0.1 mg/kg subcutaneous doses of N-803. Panels (G,H) show the corresponding Negative Log-Likelihood (NLL, Eq. 20) and Akaike Information Criterion (AICc, Eq. 21) for the Bayesian MCMC samples. Panels (I-K) show the three quality criteria, which are described in panel (F). Bayesian 95% credible intervals are marked.


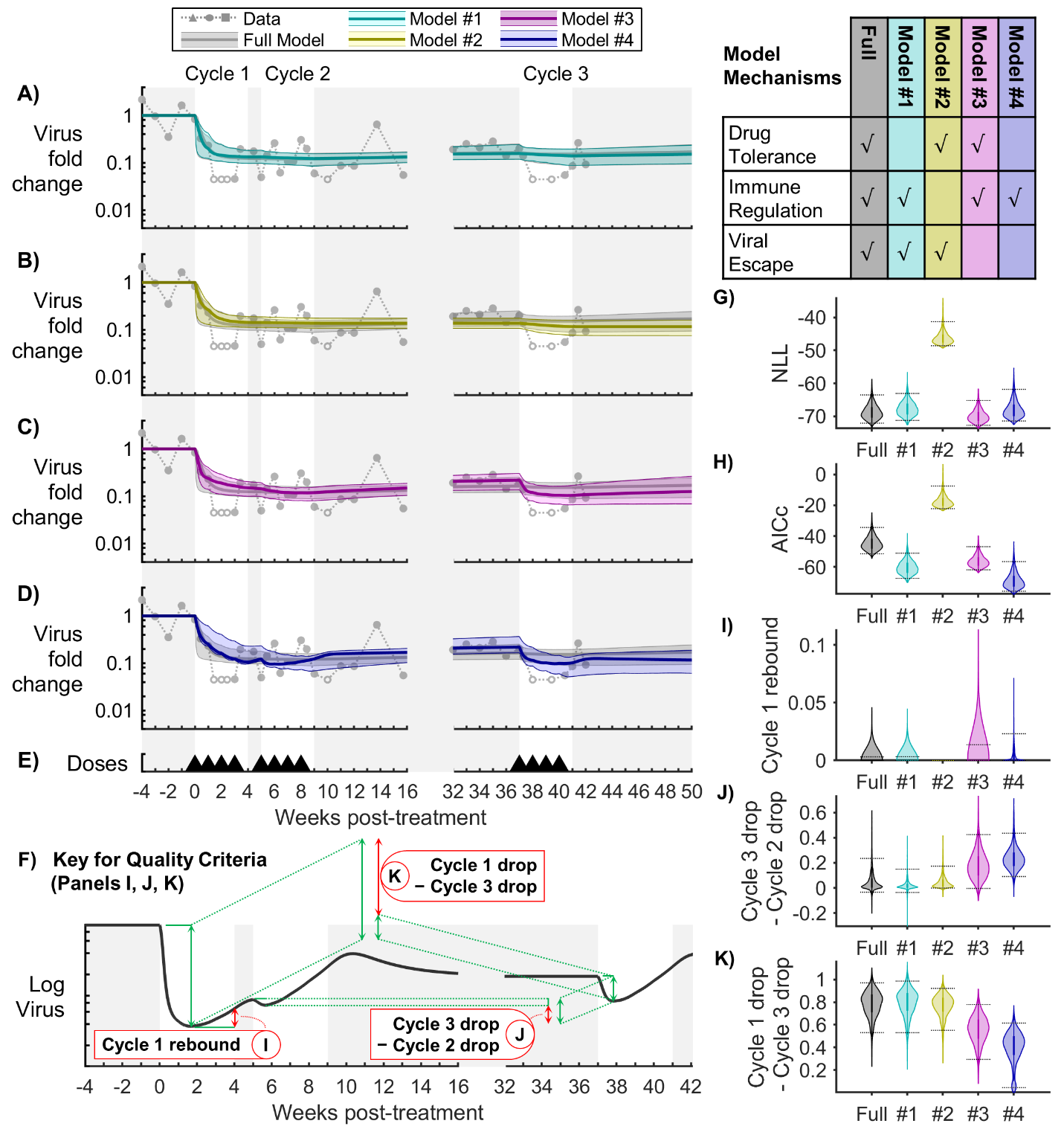


**Fig S14. Model comparison for viral load (Subject r11021).** Models with different combinations of mechanisms were compared to assess the importance of drug tolerance, immune regulation, and viral escape. Panels (A-D) compare the fold change in virus between the full model and models #1-4, respectively. The bold line corresponds to the best-fit model, and the shaded region corresponds to the Bayesian 95% credible interval. See Figure S20 for corresponding parameter distributions. Panel (E) shows timing of 0.1 mg/kg subcutaneous doses of N-803. Panels (G,H) show the corresponding Negative Log-Likelihood (NLL, Eq. 20) and Akaike Information Criterion (AICc, Eq. 21) for the Bayesian MCMC samples. Panels (I-K) show the three quality criteria, which are described in panel (F). Bayesian 95% credible intervals are marked.


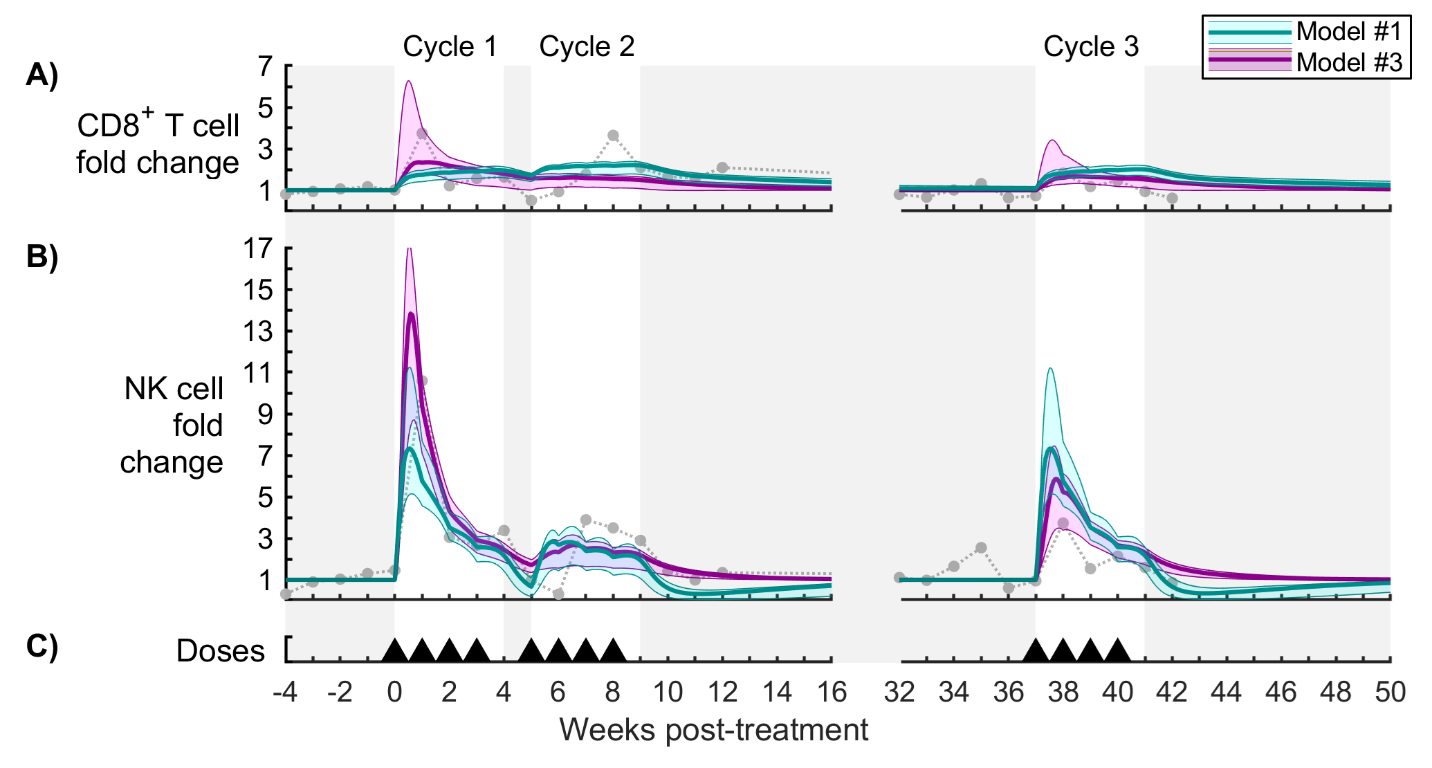


**Fig S15. Model comparison for cytotoxic cells (Subject r08016).** Panels (A,B) show fold change in CD8^+^ T cells and NK cells in the peripheral blood, respectively, for the model without drug tolerance (cyan model #1) and the model without viral escape (magenta model #3). The bold line corresponds to the best-fit model, and the shaded region corresponds to the Bayesian 95% credible interval. See Figure S18 for corresponding parameter distributions. Data from the N-803-treated SIV-infected NHP is also shown [1]. Panel (C) shows timing of 0.1 mg/kg subcutaneous doses of N-803.


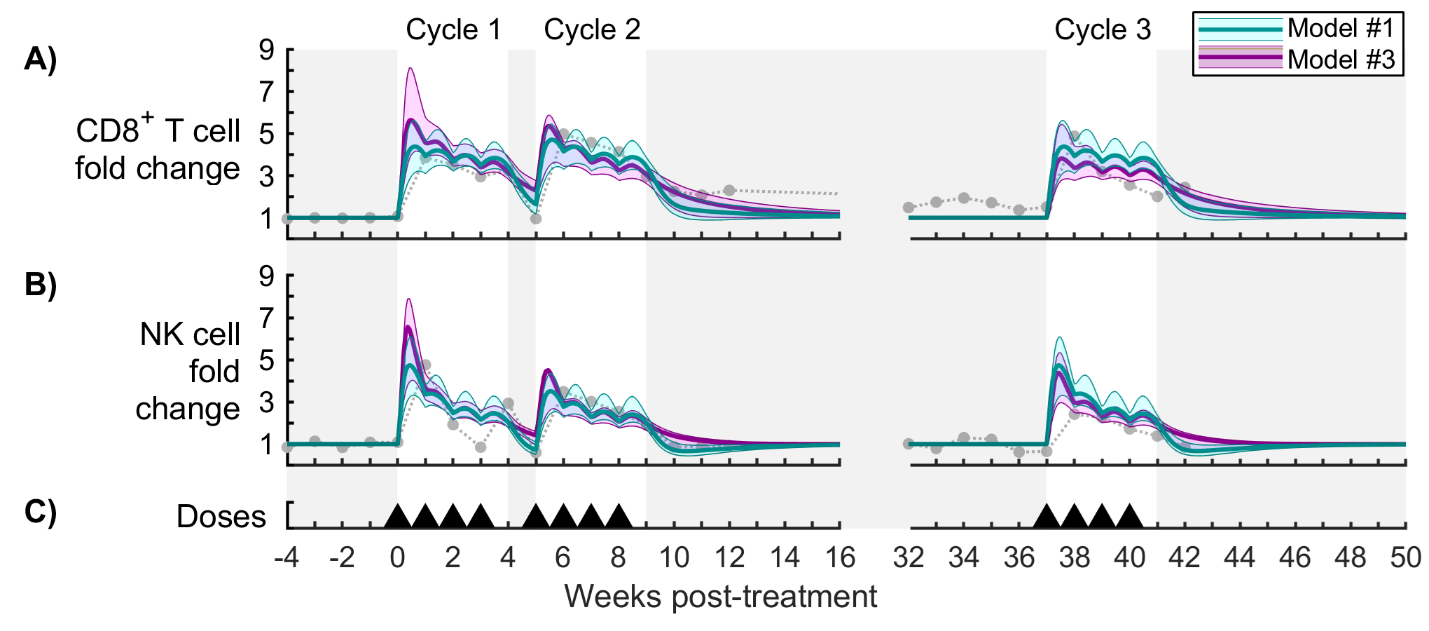


**Fig S16. Model comparison for cytotoxic cells (Subject r09089).** Panels (A,B) show fold change in CD8^+^ T cells and NK cells in the peripheral blood, respectively, for the model without drug tolerance (cyan model #1) and the model without viral escape (magenta model #3). The bold line corresponds to the best-fit model, and the shaded region corresponds to the Bayesian 95% credible interval. See Figure S19 for corresponding parameter distributions. Data from the N-803-treated SIV-infected NHP is also shown [1]. Panel (C) shows timing of 0.1 mg/kg subcutaneous doses of N-803.


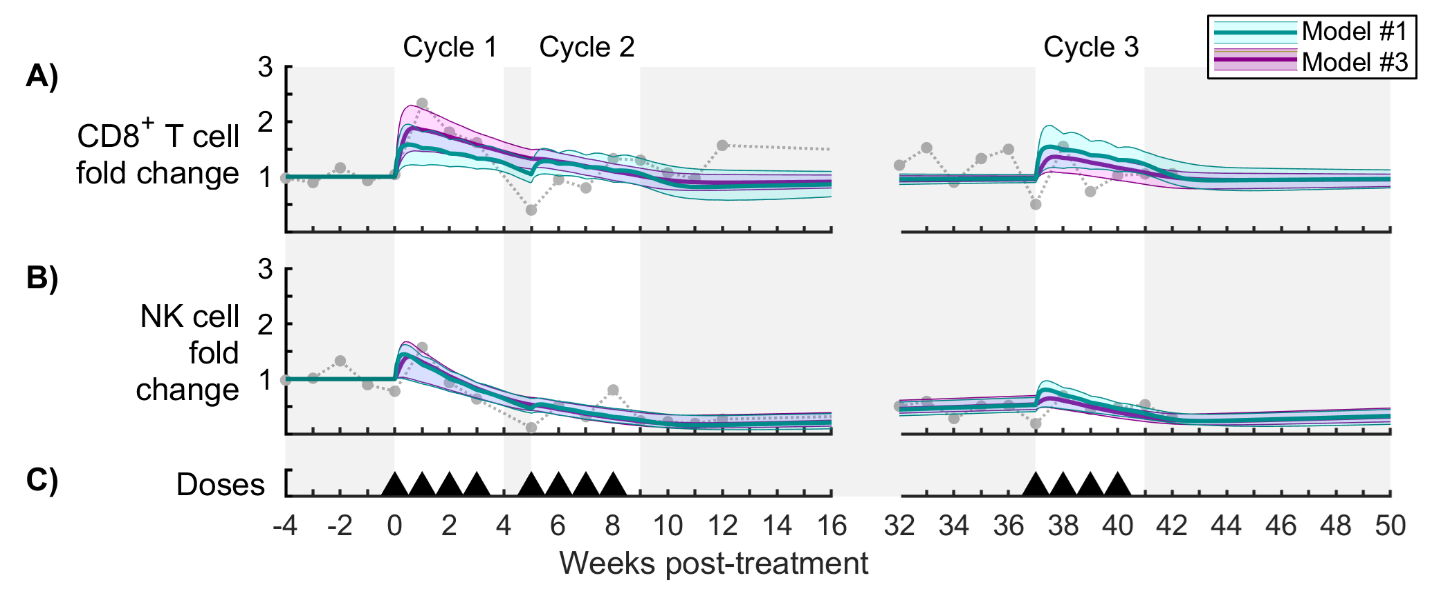


**Fig S17. Model comparison for cytotoxic cells (Subject r11021).** Panels (A,B) show fold change in CD8^+^ T cells and NK cells in the peripheral blood, respectively, for the model without drug tolerance (cyan model #1) and the model without viral escape (magenta model #3). The bold line corresponds to the best-fit model, and the shaded region corresponds to the Bayesian 95% credible interval. See Figure S20 for corresponding parameter distributions. Data from the N-803-treated SIV-infected NHP is also shown [1]. Panel (C) shows timing of 0.1 mg/kg subcutaneous doses of N-803.


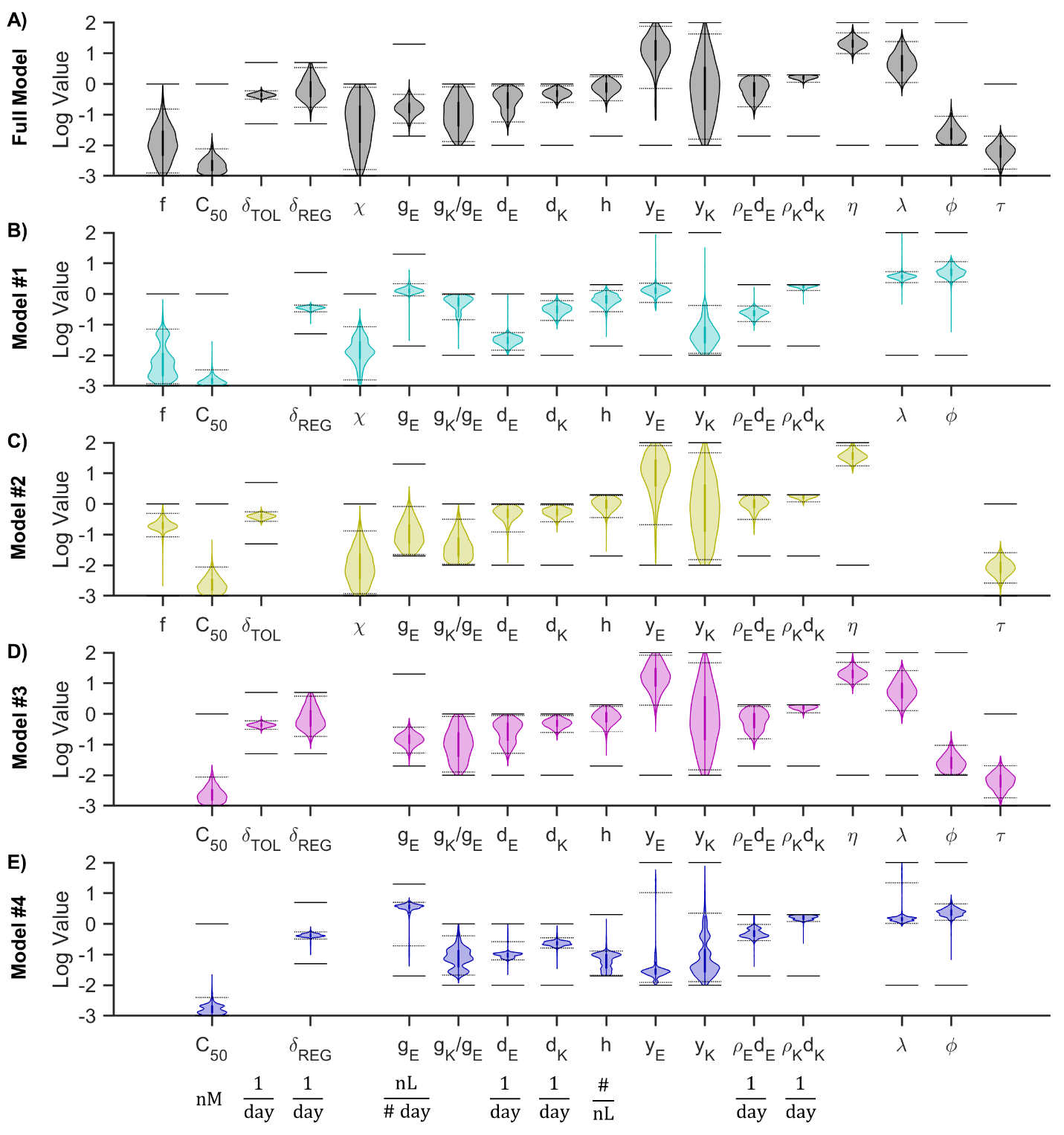


**Fig S18. Sampled parameter distributions (Subject r08016)**. Panels (A-E) show the Bayesian MCMC sample of the posterior distributions of parameter values for the full model and for models #1-4 on a logarithmic scale. Bayesian 95% credible intervals are shown as dotted lines. Allowed parameter ranges (from Table 3) are shown as solid lines. Note that some units of measurement (shown below panel E) are different from those in Table 3.


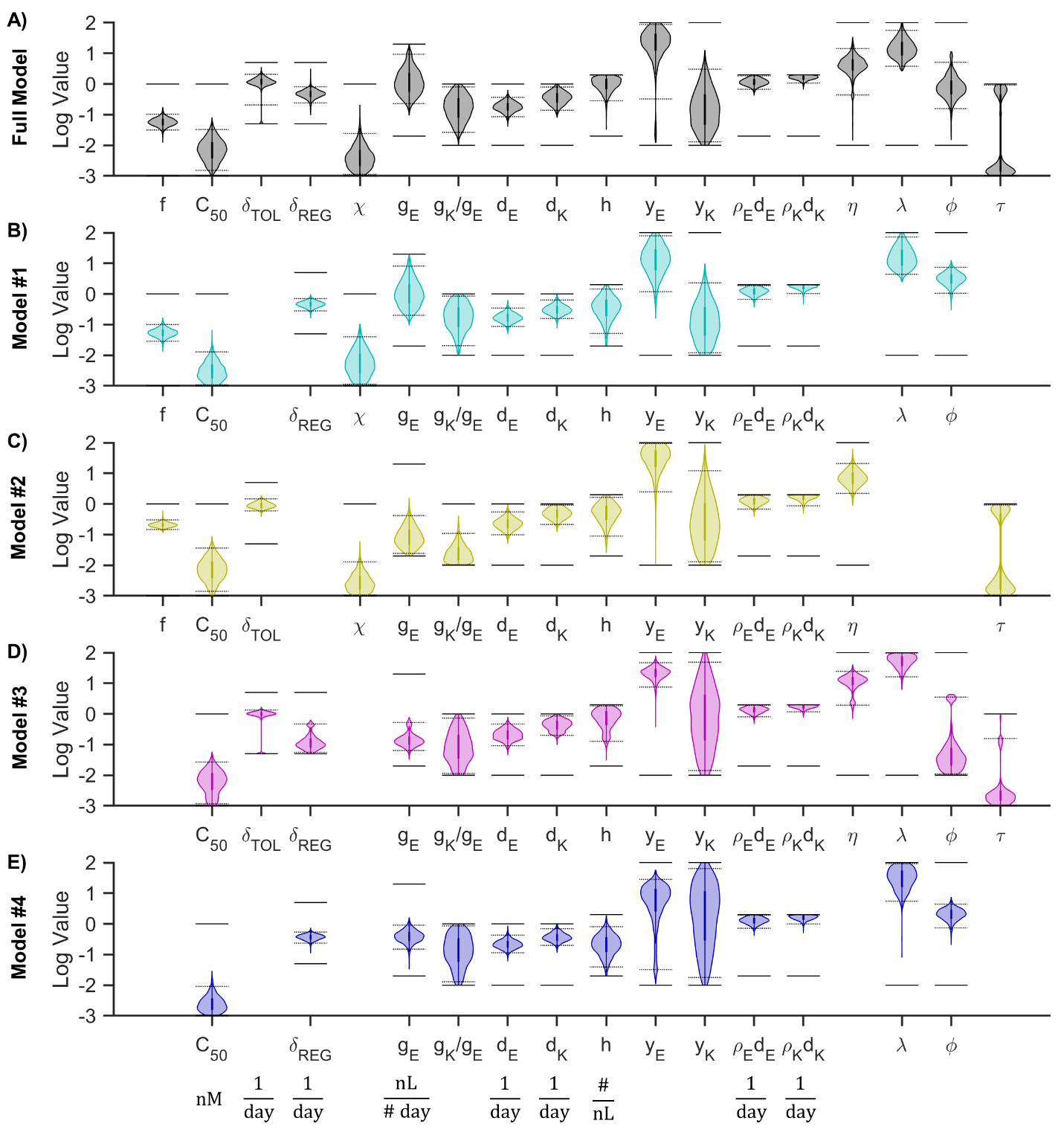


**Fig S19. Sampled parameter distributions (Subject r09089)**. Panels (A-E) show the Bayesian MCMC sample of the posterior distributions of parameter values for the full model and for models #1-4 on a logarithmic scale. Bayesian 95% credible intervals are shown as dotted lines. Allowed parameter ranges (from Table 3) are shown as solid lines. Note that some units of measurement (shown below panel E) are different from those in Table 3.


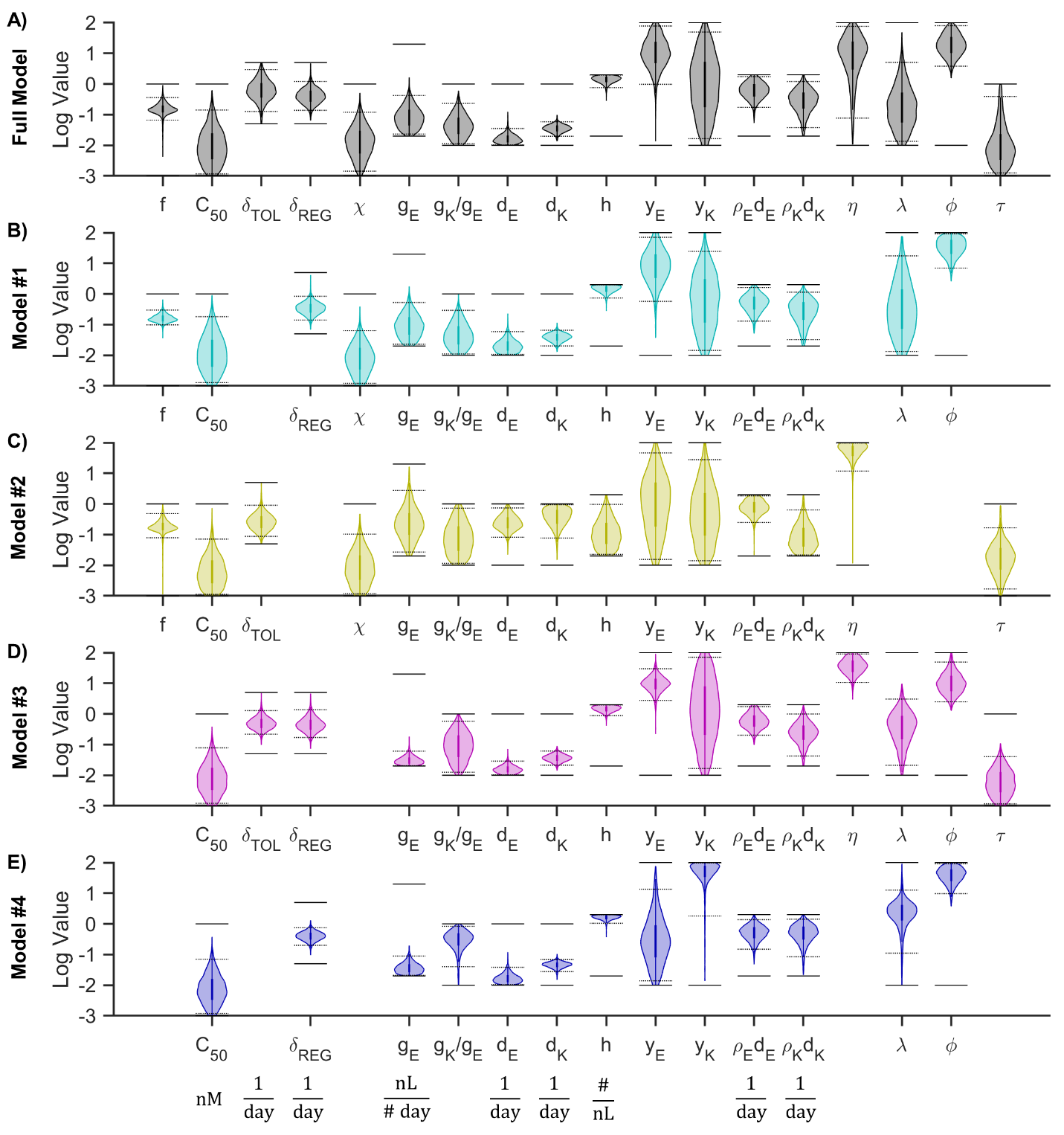


**Fig S20. Sampled parameter distributions (Subject r11021)**. Panels (A-E) show the Bayesian MCMC sample of the posterior distributions of parameter values for the full model and for models #1-4 on a logarithmic scale. Bayesian 95% credible intervals are shown as dotted lines. Allowed parameter ranges (from Table 3) are shown as solid lines. Note that some units of measurement (shown below panel E) are different from those in Table 3.

# References

1. Ellis-Connell AL, Balgeman AJ, Zarbock KR, Barry G, Weiler A, Egan JO, et al. ALT-803 Transiently Reduces Simian Immunodeficiency Virus Replication in the Absence of Antiretroviral Treatment. Journal of virology. 2018;92(3). Epub 2017/11/10. doi: 10.1128/jvi.01748-17. PubMed PMID: 29118125; PubMed Central PMCID: PMC5774892.
2. Haase AT. Population biology of HIV-1 infection: viral and CD4+ T cell demographics and dynamics in lymphatic tissues. Annual review of immunology. 1999;17:625-56. Epub 1999/06/08. doi: 10.1146/annurev.immunol.17.1.625. PubMed PMID: 10358770.
3. Ramratnam B, Bonhoeffer S, Binley J, Hurley A, Zhang L, Mittler JE, et al. Rapid production and clearance of HIV-1 and hepatitis C virus assessed by large volume plasma apheresis. Lancet (London, England). 1999;354(9192):1782-5. Epub 1999/11/30. doi: 10.1016/s0140-6736(99)02035-8. PubMed PMID: 10577640.
4. Zhang L, Dailey PJ, He T, Gettie A, Bonhoeffer S, Perelson AS, et al. Rapid clearance of simian immunodeficiency virus particles from plasma of rhesus macaques. Journal of virology. 1999;73(1):855-60. Epub 1998/12/16. PubMed PMID: 9847402; PubMed Central PMCID: PMC103903.
5. Zhang L, Dailey PJ, Gettie A, Blanchard J, Ho DD. The liver is a major organ for clearing simian immunodeficiency virus in rhesus monkeys. Journal of virology. 2002;76(10):5271-3. Epub 2002/04/23. PubMed PMID: 11967341; PubMed Central PMCID: PMC136155.
6. Cardozo EF, Andrade A, Mellors JW, Kuritzkes DR, Perelson AS, Ribeiro RM. Treatment with integrase inhibitor suggests a new interpretation of HIV RNA decay curves that reveals a subset of cells with slow integration. PLoS pathogens. 2017;13(7):e1006478. Epub 2017/07/06. doi: 10.1371/journal.ppat.1006478. PubMed PMID: 28678879; PubMed Central PMCID: PMC5513547.
7. Conway JM, Perelson AS. Residual Viremia in Treated HIV+ Individuals. PLoS computational biology. 2016;12(1):e1004677. Epub 2016/01/07. doi: 10.1371/journal.pcbi.1004677. PubMed PMID: 26735135; PubMed Central PMCID: PMC4703306.
8. Zimmermann C, Prévost-Blondel A, Blaser C, Pircher H. Kinetics of the response of naive and memory CD8 T cells to antigen: similarities and differences. European journal of immunology. 1999;29(1):284-90. Epub 1999/02/05. doi: 10.1002/(sici)1521-4141(199901)29:01<284::Aid-immu284>3.0.Co;2-c. PubMed PMID: 9933110.
9. Veiga-Fernandes H, Walter U, Bourgeois C, McLean A, Rocha B. Response of naïve and memory CD8+ T cells to antigen stimulation in vivo. Nature immunology. 2000;1(1):47-53. Epub 2001/03/23. doi: 10.1038/76907. PubMed PMID: 10881174.
10. Kim TS, Shin EC. The activation of bystander CD8(+) T cells and their roles in viral infection. Experimental & molecular medicine. 2019;51(12):1-9. Epub 2019/12/13. doi: 10.1038/s12276-019-0316-1. PubMed PMID: 31827070; PubMed Central PMCID: PMC6906361.
11. Younes SA, Freeman ML, Mudd JC, Shive CL, Reynaldi A, Panigrahi S, et al. IL-15 promotes activation and expansion of CD8+ T cells in HIV-1 infection. The Journal of clinical investigation. 2016;126(7):2745-56. Epub 2016/06/21. doi: 10.1172/jci85996. PubMed PMID: 27322062; PubMed Central PMCID: PMC4922693.
12. Bastidas S, Graw F, Smith MZ, Kuster H, Günthard HF, Oxenius A. CD8+ T cells are activated in an antigen-independent manner in HIV-infected individuals. Journal of immunology (Baltimore, Md : 1950). 2014;192(4):1732-44. Epub 2014/01/22. doi: 10.4049/jimmunol.1302027. PubMed PMID: 24446519.
13. Kim J, Chang DY, Lee HW, Lee H, Kim JH, Sung PS, et al. Innate-like Cytotoxic Function of Bystander-Activated CD8(+) T Cells Is Associated with Liver Injury in Acute Hepatitis A. Immunity. 2018;48(1):161-73.e5. Epub 2018/01/07. doi: 10.1016/j.immuni.2017.11.025. PubMed PMID: 29305140.
14. Lin JX, Leonard WJ. The Common Cytokine Receptor γ Chain Family of Cytokines. Cold Spring Harbor perspectives in biology. 2018;10(9). Epub 2017/10/19. doi: 10.1101/cshperspect.a028449. PubMed PMID: 29038115; PubMed Central PMCID: PMC6120701.
15. Au-Yeung BB, Smith GA, Mueller JL, Heyn CS, Jaszczak RG, Weiss A, et al. IL-2 Modulates the TCR Signaling Threshold for CD8 but Not CD4 T Cell Proliferation on a Single-Cell Level. Journal of immunology (Baltimore, Md : 1950). 2017;198(6):2445-56. Epub 2017/02/06. doi: 10.4049/jimmunol.1601453. PubMed PMID: 28159902; PubMed Central PMCID: PMC5340617.
16. Malek TR. The biology of interleukin-2. Annual review of immunology. 2008;26:453-79. Epub 2007/12/08. doi: 10.1146/annurev.immunol.26.021607.090357. PubMed PMID: 18062768.
17. Papillion A, Powell MD, Chisolm DA, Bachus H, Fuller MJ, Weinmann AS, et al. Inhibition of IL-2 responsiveness by IL-6 is required for the generation of GC-TFH cells. Science immunology. 2019;4(39). Epub 2019/09/15. doi: 10.1126/sciimmunol.aaw7636. PubMed PMID: 31519812; PubMed Central PMCID: PMC6820141.
18. Lugli E, Goldman CK, Perera LP, Smedley J, Pung R, Yovandich JL, et al. Transient and persistent effects of IL-15 on lymphocyte homeostasis in nonhuman primates. Blood. 2010;116(17):3238-48. Epub 2010/07/16. doi: 10.1182/blood-2010-03-275438. PubMed PMID: 20631381; PubMed Central PMCID: PMC2995354.
19. Han KP, Zhu X, Liu B, Jeng E, Kong L, Yovandich JL, et al. IL-15:IL-15 receptor alpha superagonist complex: high-level co-expression in recombinant mammalian cells, purification and characterization. Cytokine. 2011;56(3):804-10. Epub 2011/10/25. doi: 10.1016/j.cyto.2011.09.028. PubMed PMID: 22019703; PubMed Central PMCID: PMC3221918.
20. Romee R, Cooley S, Berrien-Elliott MM, Westervelt P, Verneris MR, Wagner JE, et al. First-in-human phase 1 clinical study of the IL-15 superagonist complex ALT-803 to treat relapse after transplantation. Blood. 2018;131(23):2515-27. Epub 2018/02/22. doi: 10.1182/blood-2017-12-823757. PubMed PMID: 29463563; PubMed Central PMCID: PMC5992862.
21. Rhode PR, Egan JO, Xu W, Hong H, Webb GM, Chen X, et al. Comparison of the Superagonist Complex, ALT-803, to IL15 as Cancer Immunotherapeutics in Animal Models. Cancer immunology research. 2016;4(1):49-60. Epub 2015/10/30. doi: 10.1158/2326-6066.cir-15-0093-t. PubMed PMID: 26511282; PubMed Central PMCID: PMC4703482.
22. Webb GM, Li S, Mwakalundwa G, Folkvord JM, Greene JM, Reed JS, et al. The human IL-15 superagonist ALT-803 directs SIV-specific CD8(+) T cells into B-cell follicles. Blood advances. 2018;2(2):76-84. Epub 2018/01/25. doi: 10.1182/bloodadvances.2017012971. PubMed PMID: 29365313; PubMed Central PMCID: PMC5787870 Corporation. The remaining authors declare no competing financial interests.
23. Gadhamsetty S, Beltman JB, de Boer RJ. What do mathematical models tell us about killing rates during HIV-1 infection? Immunology letters. 2015;168(1):1-6. Epub 2015/08/19. doi: 10.1016/j.imlet.2015.07.009. PubMed PMID: 26279491.
24. Jin X, Bauer DE, Tuttleton SE, Lewin S, Gettie A, Blanchard J, et al. Dramatic rise in plasma viremia after CD8(+) T cell depletion in simian immunodeficiency virus-infected macaques. The Journal of experimental medicine. 1999;189(6):991-8. Epub 1999/03/17. doi: 10.1084/jem.189.6.991. PubMed PMID: 10075982; PubMed Central PMCID: PMC2193038.
25. Choi EI, Reimann KA, Letvin NL. In vivo natural killer cell depletion during primary simian immunodeficiency virus infection in rhesus monkeys. Journal of virology. 2008;82(13):6758-61. Epub 2008/04/25. doi: 10.1128/jvi.02277-07. PubMed PMID: 18434394; PubMed Central PMCID: PMC2447079.
26. De Boer RJ, Mohri H, Ho DD, Perelson AS. Turnover rates of B cells, T cells, and NK cells in simian immunodeficiency virus-infected and uninfected rhesus macaques. Journal of immunology (Baltimore, Md : 1950). 2003;170(5):2479-87. Epub 2003/02/21. doi: 10.4049/jimmunol.170.5.2479. PubMed PMID: 12594273.
27. Davenport MP, Ribeiro RM, Perelson AS. Kinetics of virus-specific CD8+ T cells and the control of human immunodeficiency virus infection. Journal of virology. 2004;78(18):10096-103. Epub 2004/08/28. doi: 10.1128/jvi.78.18.10096-10103.2004. PubMed PMID: 15331742; PubMed Central PMCID: PMC515020.
28. McKay MD, Beckman RJ, Conover WJ. Comparison of Three Methods for Selecting Values of Input Variables in the Analysis of Output from a Computer Code. Technometrics. 1979;21(2):239-45. doi: 10.1080/00401706.1979.10489755.
29. Byrd RH, Gilbert JC, Nocedal J. A trust region method based on interior point techniques for nonlinear programming. Mathematical Programming. 2000;89(1):149-85. doi: 10.1007/PL00011391.
30. Marino S, Hogue IB, Ray CJ, Kirschner DE. A methodology for performing global uncertainty and sensitivity analysis in systems biology. Journal of theoretical biology. 2008;254(1):178-96. Epub 2008/06/24. doi: 10.1016/j.jtbi.2008.04.011. PubMed PMID: 18572196; PubMed Central PMCID: PMC2570191.
