# Supplementary material for: Mathematical modeling of N-803 treatment in SIV-infected non-human primates
Source: PLoS Comput Biol. 2021 Jul 28;17(7):e1009204. doi: 10.1371/journal.pcbi.1009204 (PMC8351941; doi:10.1371/journal.pcbi.1009204)
Supplement: Attachment — Submitted filename: Response to Reviewers.pdf [file pcbi.1009204.s009.pdf]

In addition to the reviewer-prompted changes outlined below, a few typo corrections were also made and are included in tracked changes. Note that our figure numbering has changed as a result of expanding the methods section in the main text. There is a new Figure 2 that gives the N-803 dosing. There is also a new Figure S1 that gives parameter distributions resulting from Bayesian MCMC.

We have included the requested model and experimental data in supplement spreadsheet Data S1.

Below, reviewer comments are included in quotes with our responses in bold below each comment.

Reviewer #1

"In this manuscript the authors use a quantitative model to gain new insight into mechanisms by which N-803 (an IL-15 agonist) suppresses HIV infection. The model is based on ordinary differential equations that combine the pharmacokinetics and pharmacodynamics of N-803, and is calibrated using longitudinal viral, CC8+ T cell, and NK cell measurement from the peripheral blood collected in a non- human primate (NHP) study. The model is used to assess the relative contribution of three different mechanisms that are believed to contribute to reduced N-803 treatment efficacy: 1) drug tolerance (driven by the decline in IL-15 receptor expression by CD8+ T cells); 2) immune regulation (driven by a systemic anti-inflammatory response to limit immune activation); and 3) viral escape (driven by an altered amino acid sequence of targeted CD8+ T cell epitopes during N-803 treatment). Previous studies suggest all three of these mechanisms contribute to lack of efficacy of N-803, but it has been difficult to determine the relative contribution of each. Finally, the model was used to explore theoretical treatment alterations. The model suggested that compared to drug tolerance and viral escape, immune regulation was a critical mechanism that enabled viral rebound after treatment, and that treatment could be best improved with less frequent N-803 dosing and immune regulation blockade. Overall this manuscript is well written and clear, and describes a useful quantitative tool that gives new insight into mechanisms that may be most important in N-803 efficacy. It also enables testing theoretical dosing regimens, which could be valuable for prioritizing future experiments or clinical studies."

"It would be useful to apply additional quantitative metrics and statistical tests to support some of the key conclusions, especially those in Figure 3. One of the main conclusions of the manuscript is that immune regulation is a critical mechanism that underpins viral rebound after N-803 treatment, and this is determined from treatment response curves after different mechanisms are removed from the model (Figure 3). Rather than presenting the data qualitatively in a chart (Figure 3A), these metrics could be quantified and statistical tests could be performed to support the statement. For example, is there a quantitative metric for viral rebound that is statistically different in Figure 3C compared to the other scenarios? It does appear that some of these metrics were quantified in the appendix (Fig S5, p. 10), though a direct comparison in the main text would provide added support. This could be done similarly to support conclusions in Figures 4, 5 and 6."

We have quantified the previously qualitative metrics of model behavior (see page 18, line 358 for description, Fig 4F for graphical depiction, and Eq. S1-S3 in Appendix S1). In response to another reviewer, we performed Bayesian MCMC to quantify uncertainty in model parameters and predictions. Figure 4 (previously Figure 3) has been updated to show distributions of these metrics as predicted by the Bayesian MCMC sample, and statistical tests were conducted to compare them. In addition, all model values reported in text are now reported as Bayesian 95% credible intervals.

"How could antiretroviral therapy be incorporated into the model? Could this approach be used to explore how N-803 could be effectively use without re-activating latency (McBrien at al., Science 2020). This is mentioned briefly in the discussion but could be expanded."

Per the reviewer's suggestion, we expanded discussion of how future iterations of the model could be combined with ART (page 30, line 669).

“It would be helpful to provide more detail in the symbolic legend for Figure 4 (better define both cyan and magenta lines).”

We added legends as requested in Figure 5 (previously Figure 4).

Reviewer #2:

“The authors carried out an important study to determine the contribution of an immunomodulatory IL-15 based therapy to various mechanisms affecting treatment outcomes of SIV in NHPs. The study has the potential to have high impact because it uses experimental data in combination with mechanistic mathematical models to assess biological hypotheses about the effect of the drug N-803, an IL-15 superagonist. However, as discussed in my comments below, there are several large errors in the model calibration methods that prevent the accurate interpretation of any downstream results or figures. Although all of my comments are pertinent, they are listed in order from most to least significant.”

“1) The AIC calculation in S7 in the supplemental is incorrectly applied in this study. The formula used by the authors is for univariate observations, but the models are being fit to multidimensional data. The authors need to use the multivariate AIC score found in "E.J. Bedrick and C.L. Tsai, Model selection for multivariate regression in small samples, Biometrics, 50 (1994), 226–231.". This multivariate AIC paper discuss applications to nonlinear regression models, which is the case here. The number of data points would be much less than 289 as stated in the supplemental material.”

Thank you for pointing us to a more suitable form of AIC for our model. We have updated AIC calculations per your reference and updated methods are included on page 16.

“2) Figure 2: The shaded region represents the model output from the top 20 parameter sets, as computed by the NLL. In the supplemental it says the top 20 corresponds to the top 1% of parameter sets. This is not statistically rigorous and may be somewhat misleading to the reader as a representation of statistical uncertainty in the plots. It's not clear that the shaded regions represent a 95% credible interval. If the authors want to include a shaded region, I advise that they use a Bayesian MCMC method to estimate parameters, perhaps initializing the chain from the parameter set found with the currently reported method, and then report the 95% confidence intervals on the parameters and plot the 95% credible intervals in the figure. If confidence intervals are calculated, the authors should be sure to use the correct formulation of the variance for replicate data, which is the case here because each variable is being fit to 3 replicate data sets at the same time. See for example, "Nonlinear regression" by Seber and Wild. To ensure proper evaluation of the manuscript, the authors should be clear and write the formula they used for the NLL in the supplementary material”

According to the reviewer suggestions, we sampled the posterior distribution for model parameters using a MCMC algorithm and included the resulting sample and credible intervals (Figure S1). All figures have been updated to show the 95% credible intervals in shaded regions. MCMC approach is described on page 16, line 326, and the error model and likelihood function are now given on pages 13-14 (Eq. 19-20). As a part of this change, we updated our likelihood function. Previously, we assumed independent and identical normally distributed error across all data time points, response variables, and replicates (equivalent to a univariate regression). Now, we assume separate error for each response variable with no error covariance (still identical for each replicates). We elected not to use separate error for each replicate, per Seber and Wild, because this led to a qualitatively poor fit that favored one subject over the other two (this being the subject with the most censored data and an incomplete characterization of early treatment dynamics).

“3) Related to comment 2, the authors should perform statistical uncertainty quantification, which they will obtain from a method such as Bayesian MCMC, in order to justify their choice of fixed vs. non-fixed parameters. The calculation of AIC scores does inform whether or not the model is overfit, but only allows the selection of the

"least overfit" model. Similarly, global sensitivity analysis with PRCC does not by itself provide a measure of whether the models are overfit, and therefore whether downstream conclusions can be drawn with statistical confidence."

As suggested, we conducted Bayesian uncertainty quantification as described above. Based on the resulting parameter distributions (Fig S1), we believe no further parameters could be fixed without compromising the model comparison process.

"4) In the Model Calibration in the supplemental, the authors state "Some viral data points lay on the lower limit of detection for the viral assay. For these points, error was only counted if the model value was above the data point." The authors should provide a statistical justification for this type of heuristic with the NLL function. In my opinion, these data points should just be removed from the model calibration if the authors choose to neglect them when the model solution is below the data point anyways. This would likely not effect the parameter estimation since none of the model solutions seem to drop below these data points. Otherwise, the optimization should be treated as a censored data problem."

Per the reviewer's suggestion, we omitted censored data points from consideration, and we stated such in the main text (page 14, line 298 and in Figure 3,4 captions).

Reviewer #3:

"In "Mathematical modeling of N-803 treatment in SIV-infected non-human primates" authors investigate the impact of drug tolerance, immune regulation, and viral escape in SIV viral dynamics in-host using data from non-human primate experiments with an IL-15 superagonist, N-803."

"The models presented follow CD8+ T cell and, significantly, NK cell responses in addition to make their predictions. Notably they also incorporate PK/PD of the N-803 treatment in order to gain a more accurate picture of the dynamics. They employ their model to explore alternative N-803 dosing strategies. I especially appreciate this part -- a wide range dosing strategies are impossible to test in vivo, and validated models are the perfect way to identify candidate "best" dosing strategies. The modeling is careful and the parameters thoughtfully derive from literature and explained in the extensive SI (though I would appreciate more detail in the main text, see end). I find the work intriguing but do have concerns outlined in the following."

"The authors "calibrate" the model to data. Why not use individual fitting or some other approach, e.g. nonlinear mixed effects modeling, with the longitudinal data? Is there a clear reason why this calibration approach is to be preferred?"

Given the noisiness of the data, we elected not to focus our analysis on individual fitting to avoid overinterpretation. We clarified this point in the methods (page 13, line 283). To be thorough, we repeated our procedures for each individual subject and included those results in Appendix S1 (starting page 16, Figures S9-S20). Results agreed with those presented in the main text, with the exception being the subject with the highest degree of data censoring. This is referenced in the main text (page 23, line 488) and discussed in Appendix S1. As an aside, a nonlinear mixed effects approach was explored prior to submission. However, the NLME algorithm (MATLAB's `nlmefit`) could not successfully analyze our model unless only a very small number of parameters we considered as mixed effects (e.g. initial conditions only).

"I note that the "calibrated" model does not explain model [data?] variability very well, e.g. the range in NK cell expansion (outlined in page 13), and it's also clear from the number of outliers in some of the data -- and that there are trends in the viral load data that are not captured by the model (e.g. Fig. 2a, S7a). Why? These discrepancies should be addressed and the use of the model in spite of these discrepancies should be justified."

We agree that there is considerable variation in the magnitude of responses among the three subjects. We have included a discussion of this data variability and the model's ability to reproduce this variability on page 28, line 622. In short, given the small sample size and considerable variability associated with non-human primate models, our goal was to reproduce varying response across different cycles of treatment that were separated by short or long timespans. The current model can therefore inform dosing regimens based on average population responses, and future model iterations taking into account larger samples can begin to refine the predictions for individuals with outlier responses.

"I am particularly concerned by an omission the authors identify in their discussion, of latently infected cells. Activation of provirus held in a latent state of infection is thought to be *the* driver of viral rebound following treatment interruption. Authors should justify their neglect them in an investigation of post-treatment dynamics because it seems like a massive oversight. To be clear, the statement in the discussion (page 26) on it is in my opinion inadequate, as it describes inclusion of latent cell dynamics as a potential improvement, and to me the choice of a model neglecting these dynamics as a valid tool to investigate post-treatment SIV viral dynamics must be defended and justified, up front."

We agree that our assumptions regarding latency needs to be justified as clearly and as early as possible. We added this justification to the model description (page 8, line 163). In short, the role of the latent reservoir in the response during and after immunotherapy alone remains unclear. Combined with the fact that the subjects in our experimental data were not ART-treated and the parameter uncertainty that would stem from an explicit viral reservoir in the absence of data measuring the reservoir, we opted not to explicitly account for latent viral reservoir dynamics, like other models of HIV immune therapy. Of course, we agree with the reviewer that when considering N-803 (or other immunotherapy) treatment in the context of ART and long-term suppressed viral load, mathematical models should include a representation of the latent reservoir.

"Minor points:"

"In the model derivation, dependent variables X and C are not defined. One can infer that C is drug concentration from eqs 7-8 but really they should be defined."

"Parameters lambda and phi and others are also not defined -- and yet they are particularly discussed? (page 11)."

"In the model equations (1-12) I would avoid the concentration notation. Standard models typically represent concentrations, so it's not necessary, and it's distracting."

We have made the suggested updates to clarify equation variables and improved equation presentation (page 7-12, Eq 1-14). We rearranged the model description so that variables and parameters are defined immediately following each equation.

"Authors create a delay in drug tolerance via a chain of ODEs, giving an Erlang-distribution on drug tolerance in time. Could graph resulting distribution. If there were some data one could compare the distribution to, visually, would lend further support for model choices."

We altered our supplemental Figure S8 (Appendix S1, page 11) to compare the regulation and tolerance variables to experimental indicators. We reference this in the main text.

"Authors define a per-cell killing verbally (page 16) but do not explain how it is computed in the main text. Which is frustrating as a reader, because they then rely on it to interpret results."

We included PCK and related equations in the main text (Eq. 22-30, page 18-19).

“A detail I perhaps missed, why are CTL and NK cell populations not stimulated by foreign pathogen?”

We have included clarification regarding our assumption of constant CTL and NK cell stimulation in the model description. (page 9, line 191).

“Linking together most minor comments: I would encourage revision of the methods in the main text to make it "stand alone" better. For example, "calibration" is not a well-defined process, that should be briefly outlined for the reader, who then can go to the SI for more details - otherwise the "ranges" in parameters and results comes out of nowhere. Also an indication of parameters were calibrated vs taken from literature to help with a critical reading from this study should be in the main text. Another example: I found this sentence in the SI, "Modifications to killing rate via drug stimulation, drug tolerance, and immune regulation (Eqs. 7,8) represented changes in both the frequency of cytotoxically active cells within their respective total populations and changes in the individual efficacy cytotoxically active cells." It's an important assumption for model implementation, and not at all clear from the main text.”

We moved the majority of the methods description into the main text. This included a parameter table (Table 3, page 14), equations for calculated parameters (Eq. 15-18, page 12) and per-cell killing, and descriptions of procedures for parameter estimation (page 13), uncertainty quantification, and model comparison (page 16).
